# Paeoniflorin-loaded MBG nanogel alleviates oxidative microenvironment and reinforces subchondral bone regeneration for osteoarthritis treatment

**DOI:** 10.1093/rb/rbag016

**Published:** 2026-02-05

**Authors:** Zugui Wu, Xiuhong Huang, Jiao Li, Gaoquan Zheng, Feng Peng, Wei Dong, Yue Zhu, Yu Zhang, Rong Yuan, Zhiwei Wu, Ying Guo, Jin Xiao, Biaolin Wan

**Affiliations:** The Third Clinical College of Yunnan University of Chinese Medicine, The Third Affiliated Hospital of Yunnan University of Chinese Medicine, Kunming 650500, China; Guangdong Cardiovascular Institute, Guangdong Provincial People’s Hospital (Guangdong Academy of Medical Sciences), Southern Medical University, Guangzhou 510080, China; School of Basic Medical Sciences, Guangzhou University of Chinese Medicine, Guangzhou 510006, China; The Third Clinical College of Yunnan University of Chinese Medicine, The Third Affiliated Hospital of Yunnan University of Chinese Medicine, Kunming 650500, China; Medical Research Institute, Department of Orthopaedics, Guangdong Provincial People’s Hospital (Guangdong Academy of Medical Sciences), Southern Medical University, Guangzhou 510080, China; Medical Research Institute, Department of Orthopaedics, Guangdong Provincial People’s Hospital (Guangdong Academy of Medical Sciences), Southern Medical University, Guangzhou 510080, China; The Third Clinical College of Yunnan University of Chinese Medicine, The Third Affiliated Hospital of Yunnan University of Chinese Medicine, Kunming 650500, China; The Third Clinical College of Yunnan University of Chinese Medicine, The Third Affiliated Hospital of Yunnan University of Chinese Medicine, Kunming 650500, China; Medical Research Institute, Department of Orthopaedics, Guangdong Provincial People’s Hospital (Guangdong Academy of Medical Sciences), Southern Medical University, Guangzhou 510080, China; The Third Clinical College of Yunnan University of Chinese Medicine, The Third Affiliated Hospital of Yunnan University of Chinese Medicine, Kunming 650500, China; The Third Clinical College of Yunnan University of Chinese Medicine, The Third Affiliated Hospital of Yunnan University of Chinese Medicine, Kunming 650500, China; The Third Clinical College of Yunnan University of Chinese Medicine, The Third Affiliated Hospital of Yunnan University of Chinese Medicine, Kunming 650500, China; Medical Research Institute, Department of Orthopaedics, Guangdong Provincial People’s Hospital (Guangdong Academy of Medical Sciences), Southern Medical University, Guangzhou 510080, China; Department of Joint Surgery and Sports Medicine, Heyuan People’s Hospital, Guangdong Provincial People’s Hospital Heyuan Hospital, Heyuan 517100, China

**Keywords:** paeoniflorin, ROS scavenging, osteoarthritis, articular cartilage, subchondral bone, nanogel

## Abstract

Osteoarthritis (OA) involves synergistic pathological changes in both cartilage degradation and subchondral bone remodeling due to oxidative stress, yet current therapies typically target these processes separately. To address this limitation, we developed a dual-functional nanogel (MBG@Pae@HA) by conjugating hyaluronic acid (HA) and paeoniflorin (Pae) to mesoporous bioactive glass nanoparticles via amide bonding and non-covalent interactions, respectively. Leveraging the inherent hydrophilicity of HA, MBG@Pae@HA spontaneously forms nanogels and shows enhanced adhesion force which ensure extended Pae and bioactive ions release in the OA microenvironment. MBG@Pae@HA exhibits potent reactive oxygen species (ROS)-scavenging capacity, contributing to the homeostatic balance between cartilage matrix anabolism and catabolism. Transcriptomic analysis revealed that MBG@Pae@HA treatment reactivates chondrocyte function through activating the cAMP/PKA/CREB antioxidant pathway. Furthermore, with controlled release of Pae and bioactive components (including Ca ions, Si ions and ${\text{PO}}_{4}^{3-}$) it significantly promotes osteogenesis of bone stem cells. Medial meniscotibial ligament (DMM) model demonstrated the dual therapeutic efficacy of MBG@Pae@HA nanogel with histological and micro-CT analyses confirming concurrent protection against cartilage degradation and enhancement of subchondral bone regeneration. By simultaneously addressing both major pathological features of OA through microenvironment remodeling and prolonged drug delivery, this HA-functionalized and Pae-loaded nanogel represents a significant advance in OA treatment strategies, offering a comprehensive approach to halt disease progression and promote joint repair.

## Graphical Abstract

**Graphical Abstract:**
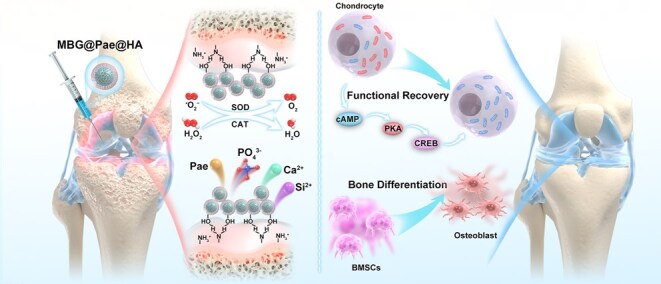

## Introduction 

Osteoarthritis (OA) is a prevalent degenerative joint disease, which affects 44.7% of patients with risk of disability [1]. The hallmark pathological features include progressive cartilage degradation, accompanied by synovial inflammation that drive chondrocyte dysfunction, extracellular matrix (ECM) degradation, osteophyte formation and subchondral bone remodeling [2–4]. This vicious cycle disrupts chondrocyte homeostasis, accelerating cartilage destruction and disease progression. Current nonoperative therapies primarily focus on symptomatic pain relief to preserve residual joint function, yet fail to halt or reverse underlying disease progression [5, 6].

A central mediator of OA processes is reactive oxygen species (ROS), which not only exacerbate cartilage breakdown but also amplify synovial inflammation [7, 8]. Mitochondrial dysfunction plays a pivotal role in OA pathogenesis [9–11]. Impaired mitochondrial membrane potential leads to excessive ROS generation, establishing a self-perpetuating cycle: oxidative stress further damages mitochondria, depletes cellular ATP and ultimately activates apoptotic pathways. Paeoniflorin (Pae), derived from paeonia lactiflora, is a model drug for OA treatment [12, 13]. Pae exhibits anti-inflammatory [14], antioxidant [15] and chondroprotective effects [13], effectively suppressing key inflammatory mediators (e.g. ROS, IL-1β) and inhibiting ECM degradation in chondrocytes. However, relying solely on traditional Chinese medicine is difficult to exert a long-term effect in harsh micro-environments.

Mesoporous bioactive glass (MBG) is characterized by a three-dimensional network of covalently bonded SiO_4_ tetrahedra, incorporating soluble modifier ions (e.g. Ca^2+^, Na^+^, ${\text{PO}}_{4}^{3-}$) [16]. Through sol-gel processing, MBG exhibits controlled dissolution in physiological environments, forming single-component bioactive particles [17, 18]. Notably, MBG demonstrates excellent biocompatibility and metabolically inert degradation that also serve as biomineralization precursors, promoting bone regeneration [19]. MBGs feature highly ordered 5–20 nm mesopores, large pore volume and superior surface area versus conventional bioglasses, enabling exceptional drug loading and controlled ion release for enhanced therapeutic efficacy [20, 21]. Owing to these favorable properties, MBG functions as an effective drug delivery platform for targeted OA therapy.

While subchondral bone remodeling is considered, joint surface lubrication remains a key therapeutic strategy for symptom relief. A promising approach employs macromolecular complexes such as hyaluronic acid (HA) as natural boundary lubricants on cartilage surfaces [22]. These complexes interact with cartilage lipids, forming a hydration layer that reduces friction, aiding in joint wear improvement in OA patients [23]. Conventional clinical strategies employ intra-articular corticosteroid and HA injections to restore joint lubrication, though their efficacy is limited by short duration and need for repeated administration [24, 25]. To address these limitations, cartilage-targeting graft copolymers have been engineered to specifically bind exposed amino groups on degenerated cartilage for sustained OA therapy [26–28]. However, their ability to modulate the inflammatory microenvironment and enhance ECM remodeling remains suboptimal. The MBG sustained-release system offers a promising solution to this therapeutic challenge.

**Figure 1:**
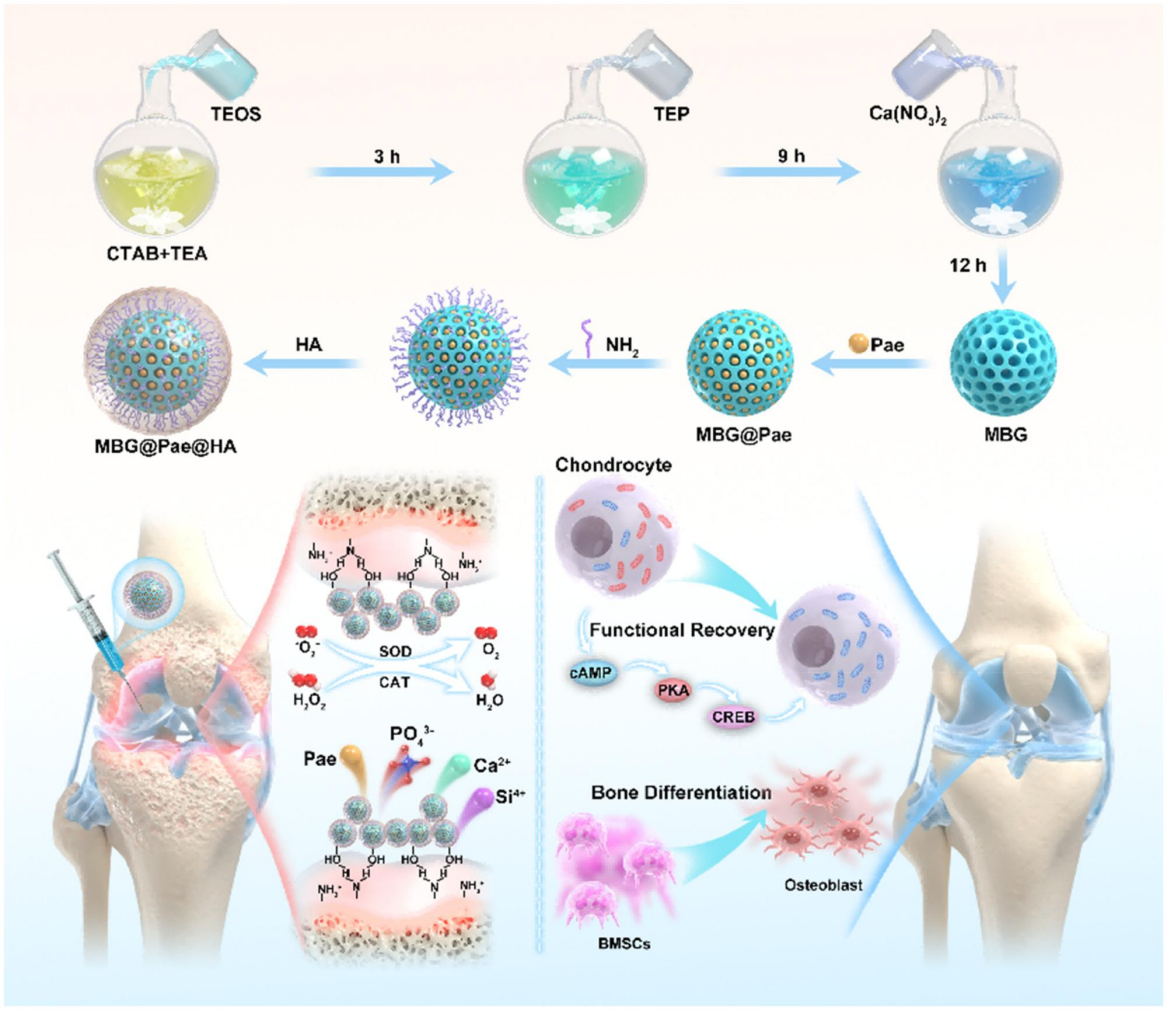
Schematic of the synthesis process and application model of MBG@Pae@HA. MBG is synthesized via template synthesis to load Pae (MBG@Pae), following coating HA on the surface of MBG@Pae@HA. MBG@Pae@HA is injected into the joint cavity. During joint activity, the HA-rich hydroxyl groups form hydrogen bonds with water molecules, leading to the aggregation of nanogel. The therapeutic efficacy and mechanistic basis of MBG@Pae@HA in osteoarthritis through chondrocyte-targeted reactive oxygen species scavenging and subchondral bone repair.

Here, we proposed a MBG-based nanogel by functionalization of HA via amide reaction on the surface of Paeoniflorin-loaded MBG (denoted as MBG@Pae@HA) (Figure 1). The hydroxyl group of MBG@Pae can create hydrogen bonds with the body fluid environment under physiological conditions, reinforcing the structure of nanogel. The adhesion capability of MBG@Pae increased after coating HA, allowing MBG@Pae@HA to adhere well to the damaged cartilage surface. The findings demonstrate that MBG@Pae@HA, functioning as a highly efficient and biocompatible ROS scavenger, effectively alleviates articular inflammatory responses in chondrocytes. Specifically, the released HA enhances surface adhesion and provides lubrication, while Pae exerts antioxidant effects to remodel the immune microenvironment. The bioactive ions (Si, Ca, ${\text{PO}}_{4}^{3-}$) promote osteogenesis through ionic stimulation. Transcriptome sequencing revealed the mechanism of MBG@Pae@HA in reinforces subchondral bone regeneration by activating cAMP-PKA-CREB signaling pathways. Animal experiments have further demonstrated that the functional nanogel effectively inhibited the progression of OA and facilitated cartilage repair, achieving the reversal of OA progression. This study confirms that MBG@Pae@HA, as a high-efficiency disease-modifying nanoplatform for osteoarthritis therapy, can effectively halt or even reverse osteoarthritis progression by simultaneously scavenging intracellular reactive oxygen species and promoting subchondral bone regeneration.

## Results and discussion

### Synthesis and characterization of paeoniflorin-loaded MBG nanogel (MBG@Pae@HA)

**Figure 2:**
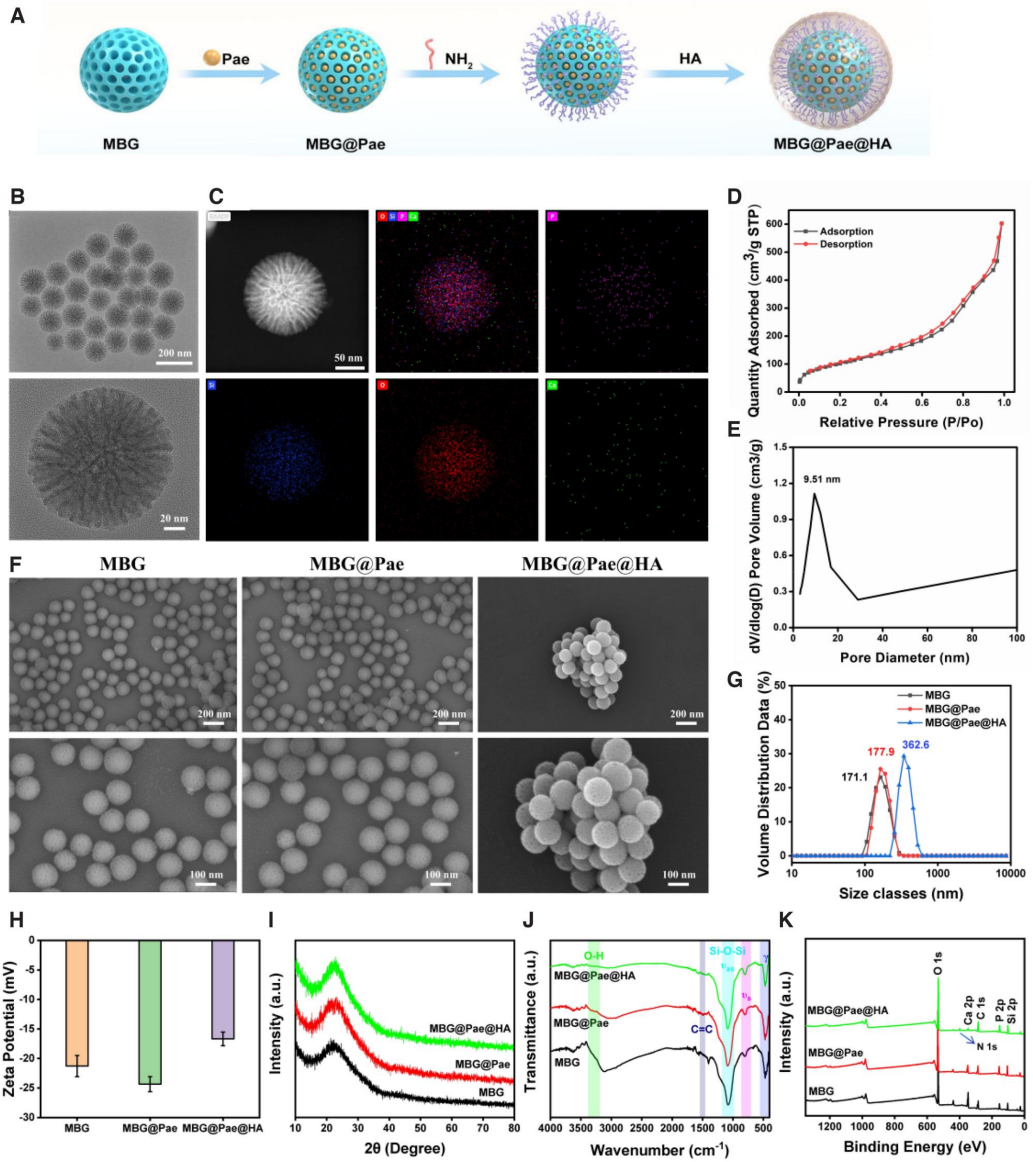
(**A**) Schematic of the synthesis process of MBG@Pae@HA. (**B**) TEM images and (**C**) EDS analysis of MBG. (**D**) N_2_ adsorption isotherm and (**E**) pore volume of MBG. (**F**) SEM images of MBG, MBG@Pae and MBG@Pae@HA. (**G**) Pore size distribution, (**H**) Zeta potential, (**I**) XRD pattern, (**J**) FTIR and (**K**) XPS survey of MBG, MBG@Pae and MBG@Pae@HA.

The synthesis steps of the MBG@Pae@HA nanocatalytic reactor were shown in Figure 2A. The TEM images in Figure 2B revealed that MBG particles were uniformly dispersed and exhibited a partially ordered mesoporous architecture. The element mapping of MBG (Figure 2C) demonstrated the homogenous distribution of P, Si, O and Ca elements. Nitrogen adsorption–desorption isotherm analysis (Figure 2D) showed that the isotherm corresponded to a type IV curve with an H1 hysteresis loop, indicating that an ordered mesoporous structure with a narrow pore size distribution [29]. The pronounced hysteresis loop in the mid-to-high pressure region confirms the existence of mesopores. Figure 2E displayed the pore size distribution curve, the peak at 9.51 nm corresponds to the most probable pore size, demonstrating a well-defined mesoporous structure with narrow size distribution.

The SEM results (Figure 2F) revealed that MBG, MBG@Pae and MBG@Pae@HA had uniform spherical shape (diameter 100–200 nm). MBG displays uniform spherical morphology with radially oriented mesopores (consistent with Figure 2B and E), and the porous structure was clearly visible at higher magnification. MBG@Pae and MBG@Pae@HA retain the spherical framework, and MBG@Pae@HA aggregate together, indicating successful HA coating forms a thin layer over the MBG@Pae surface. The retained spherical morphology postmodification suggests good structural stability during loading and coating processes.

Figure 2G showed that the pristine MBG microspheres display a narrow and monodisperse size distribution, with the majority of particles falling around 171.1 nm, indicative of their well-defined spherical morphology and uniform mesoporous structure. Upon loading with Pae (MBG@Pae), the distribution curve shifts slightly toward larger sizes (177.9 nm), suggesting successful drug encapsulation within the mesopores that may lead to minimal particle aggregation or pore expansion while maintaining structural integrity. The subsequent coating with HA (MBG@Pae@HA) results in a more pronounced rightward shift and a broader size distribution (362.6 nm), clearly demonstrating the formation of an additional surface layer that increases the overall particle diameter. This stepwise increase in particle size—from the unmodified MBG to the drug-loaded and HA-coated counterparts—confirms the successful sequential modification of the microspheres while preserving their fundamental architecture. The controlled enlargement and tailored size distribution of the final MBG@Pae@HA composite highlight its potential as a versatile drug delivery platform, where the optimized particle size can enhance circulation time and target-specific accumulation for improved therapeutic efficacy [30].

As shown in Figure 2H, the MBG microspheres exhibited a stable negative zeta potential of approximately −20 to −25 mV, originating from the dissociation of Si-OH in the mesoporous silica framework. Upon loading with Pae to form MBG@Pae, the potential remained around −25 mV. Subsequent coating with HA resulted in MBG@Pae@HA showing an increased potential of −15 to −20 mV, indicating reduced electronegativity. This phenomenon may be attributed to the steric hindrance effect of HA polymer chains—although containing numerous carboxylate groups (-COO^-^), the bulky hydrophilic coating causes an outward shift of the shear plane during dynamic light scattering measurements, leading to decreased absolute zeta potential values [31, 32]. Additionally, the flexible conformation of HA chains may partially fold and shield the charged functional groups.

To assess whether Pae encapsulation alters the crystal structure of MBG, we analyzed the components and structure of MBG@Pae@HA. The X-ray diffraction (XRD) patterns in Figure 2I revealed that MBG, MBG@Pae and MBG@Pae@HA exhibit a broad amorphous halo centered at ∼22° (2θ), confirming the non-crystalline nature of the mesoporous silica framework in MBG. No sharp crystalline peaks were observed in MBG@Pae, indicating that Pae is uniformly dispersed in an amorphous state within the mesopores without phase separation. For MBG@Pae@HA, the maintained broad hump with slight intensity variations suggests that the HA coating does not alter the amorphous structure of the underlying MBG@Pae composite. The absence of new diffraction peaks in MBG@Pae@HA further verifies that HA forms a noncrystalline surface layer, consistent with its typical polymeric amorphous nature. The Fourier transform infrared spectroscopy (FTIR) spectra in Figure 2J exhibited characteristic silica framework vibrations, including the broad Si-O-Si asymmetric stretch (1000–1100 cm^−1^), Si-OH/P-O stretching vibration (960 cm^−1^), symmetric stretching vibration of Si-O-Si (∼800 cm^−1^) and Si-O/P-O bending vibration (∼500 cm^−1^). Upon Pae loading (MBG@Pae) and the subsequent HA coating (MBG@Pae@HA), the above characteristic peaks of MBG have not changed significantly. Additionally, intensity of the peaks emerged at 1500 cm^−1^ (C = C aromatic stretches) and 3200–3500 cm^−1^ (O-H stretches) were markedly different in MBG@Pae and MBG@Pae@HA, confirming the successful introduction of carboxylate signatures and broad hydroxyl stretching vibrations. Notably, the slight broadening of the Si-OH band in MBG@Pae suggests hydrogen bonding between Pae and silanols, while the stable aromatic C = O positions in MBG@Pae@HA prove Pae’s chemical stability during HA encapsulation. The results demonstrated that MBG@Pae@HA retained both the Pae-loading signature and silica peaks, confirming a non-disruptive surface modification with HA. FTIR spectra revealed that Pae was encapsulated within MBG rather than loaded on the surface. The X-ray photoelectron spectroscopy (XPS) analysis (Figure 2K) revealed the characteristic SiO_2_ signatures of MBG at 103.5 eV (Si 2p) and 533.2 eV (O 1 s), along with the peak at 132–136 eV (P 2p), ∼350 eV (Ca 2p). Upon sequential modification with Pae (MBG@Pae) and HA coating (MBG@Pae@HA), the emergence of the above peak have minor offset (Supplementary Figures S1–S3). Notably, the minimal binding energy shift of Si 2p indicates excellent structural integrity of the SiO_2_ framework throughout the modification process. Furthermore, a clear N 1 s signal appears in the spectra of MBG@Pae@HA is consistent with the presence of amino/amide-type nitrogen species on the particle surface and is in line with the expected reaction between the surface –NH_2_ groups introduced by APTES and the –COOH groups of HA. Overall, the results in Figure 2B–K confirmed that Pae was not involved in coordination but was physically confined within the mesoporous structure of MBG. Thus, the interactions between Pae and MBG are dominated by non-covalent forces, mainly physical adsorption and hydrogen bonding, without the formation of new covalent bonds. These results confirm the successful stepwise assembly of the drug delivery system while preserving the bioactivity of HA carboxyl groups—a prerequisite for targeted applications. The organic solvent-based loading strategy minimizes premature drug leakage. In addition, the release profiles of Ca and Si ions from various groups are shown in Supplementary Figure S4. MBG exhibits the highest Ca release, while MBG@Pae shows a slightly reduced but comparable release (Supplementary Figure S4a). The HA-coated MBG@Pae@HA displays a slower and lower Ca release, indicating that the HA shell provides a diffusion barrier and enables more sustained ion release. A similar trend is observed for Si ions in Supplementary Figure S4b. These results confirm that all three materials provide continuous Ca and Si ion release in a moderate concentration range.

**Figure 3:**
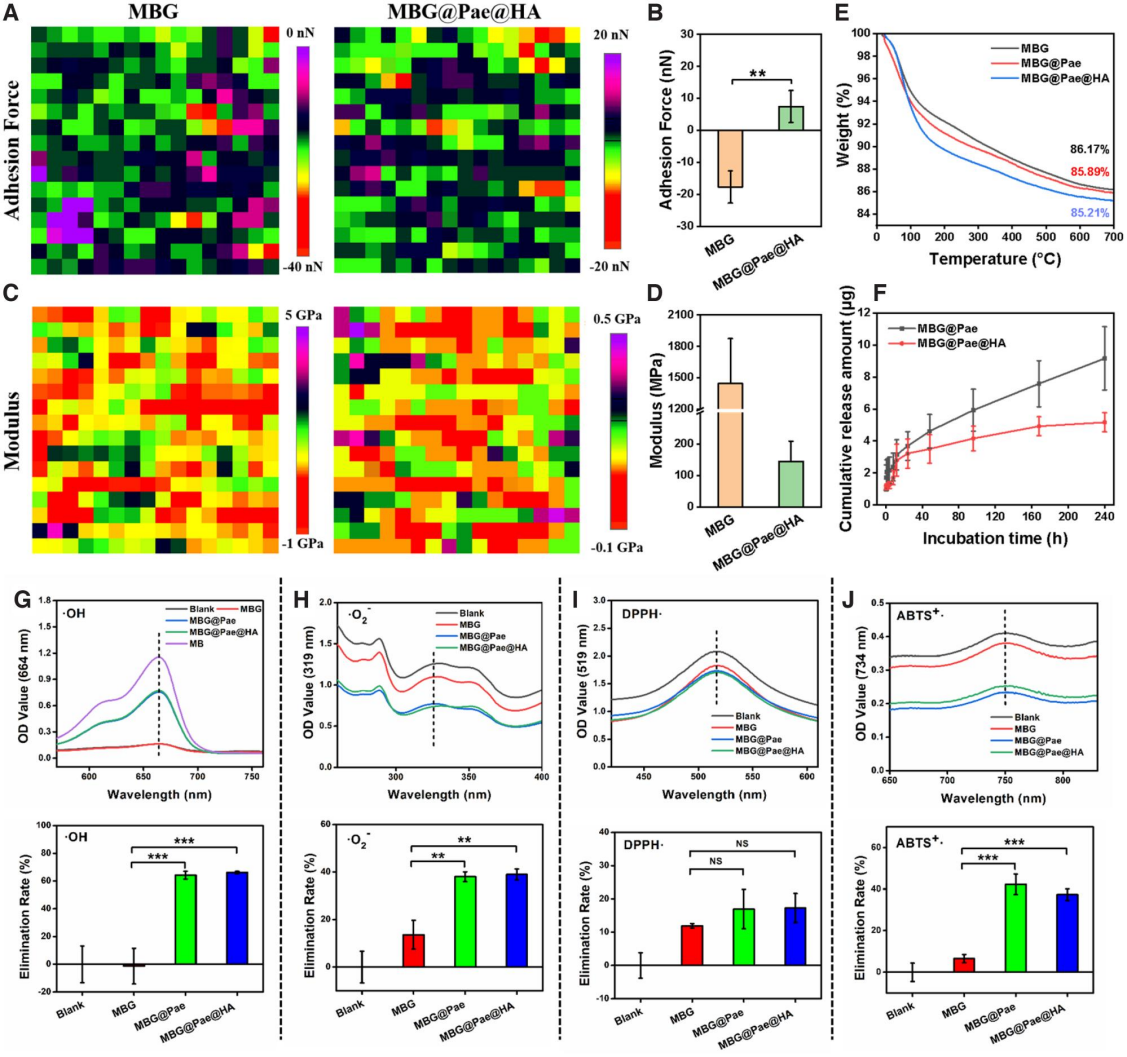
(**A**) Heatmaps and (**B**) relative frequency histograms of adhesion forces of MBG and MBG@Pae@HA. (**C**, **D**) The Young’s modulus of MBG and MBG@Pae@HA. (**E**) TGA of MBG, MBG@Pae and MBG@Pae@HA. (**F**) Cumulative mass variation of Pae release behaviors. (**G–J**) The ROS scavenging activity of the MBG@Pae@HA: (**G**)**·**OH, (**H**)**·**$O_{2}^{-}$, (**I**) DPPH**·** and (**J**) ABTS^+^**·** and their corresponding quantitative analysis.

Collectively, we have successfully synthesized MBG@Pae@HA through a template synthesis approach. Concurrently, we demonstrated that the Pae was encapsulated internally within the MBG structure rather than loaded on the surface, which is expected to greatly enhance the stability of the drug. Additionally, our study quantitatively analyzed the adhesion force dynamics and Young’s modulus of MBG and MBG@Pae@HA. As delineated in Figure 3A and B, the adhesion force exhibited by MBG@Pae@HA surpasses that of MBG, underscoring its heightened capability to adhere to and engage with the cartilage surface post-injection into the joint cavity. The average Young’s modulus of MBG registers at 1400 MPa, contrasting with the 150 MPa exhibited by MBG@Pae@HA (Figure 3C and D). This discernible reduction in rigidity signifies the formation of a nanogel structure by MBG@Pae@HA, characterized by its notably decreased stiffness. Such diminished rigidity confers distinct advantages, facilitating optimal placement within the intricate confines of the joint cavity and fostering enhanced interaction with the surrounding tissues.

Thermogravimetric analysis (TGA) can reflect the Pae loading efficiency, HA coating content and thermal stability of MBG@Pae@HA (Figure 3E). The pristine MBG exhibited a residual mass of 86.17%, while Pae loading reduced this to 85.89% in MBG@Pae, corresponding to a drug loading efficiency of approximately 0.28%. Subsequent HA coating (MBG@Pae@HA) showed negligible impact on the residual weight (85.21%), indicating the HA layer constituted less than 1% of the total mass while maintaining the structural integrity of the drug-loaded system. The initial weight loss below 100°C was attributed to moisture evaporation, followed by the decomposition of organic components (Pae and HA) at higher temperatures, with the remaining weight representing the thermally stable MBG inorganic framework. Additionally, the minimal mass loss (<15%) across all samples below 700°C suggests excellent thermal stability of the composite system.

The drug release profiles in Figure 3F demonstrated distinct kinetic behaviors between the two formulations. MBG@Pae exhibits a sharply release within 24 h followed by sustained release, reaching high cumulative release at 240 h, characteristic of diffusion-dominated release from mesopores. In contrast, MBG@Pae@HA effectively decreased the initial burst, with exhibit sustained-release properties within 240 h, indicating successful HA coating functionality as a diffusion barrier. The HA coating effectively modulates drug release kinetics while maintaining payload integrity, critical for achieving spatiotemporal control in targeted drug delivery applications.

### Free-radical scavenging capabilities of MBG@Pae@HA

The pathogenesis of osteoarthritis is strongly associated with excessive ROS production and the subsequent cellular oxidative stress damage [33]. Accordingly, the free radical scavenging activity of MBG@Pae@HA was systematically evaluated. The quantified **·**OH scavenging efficiency of MBG@Pae and MBG@Pae@HA reached over 60% (Figure 3G). Furthermore, the representative intracellular ROS, O_2_**·**^−^ were also chosen in the evaluation of MBG@Pae@HA against ROS [34]. The UV−Vis signal of the characteristic peaks of O_2_**·**^−^ diminished dramatically after the addition of MBG@Pae and MBG@Pae@HA (Figure 3H). Upon Pae loaded, MBG@Pae and MBG@Pae@HA could efficiently scavenge DPPH**·** compared to the blank group (Figure 3I), and similar result was detected in the ABTS assay (Figure 3J), thereby indicating the marvelous ROS-scavenging capability of the MBG@Pae and MBG@Pae@HA [35, 36]. In all, the ROS scavenging abilities of MBG@Pae and MBG@Pae@HA were both better than that of MBG, while there was no difference in the ROS scavenging performance between groups MBG@Pae and MBG@Pae@HA. This indicates that Pae has the ability to scavenge ROS. Collectively, these findings validate that MBG@Pae@HA functions as a potent antioxidant platform.

### *In vitro* ROS-protective effect of MBG@Pae@HA on chondrocytes

**Figure 4:**
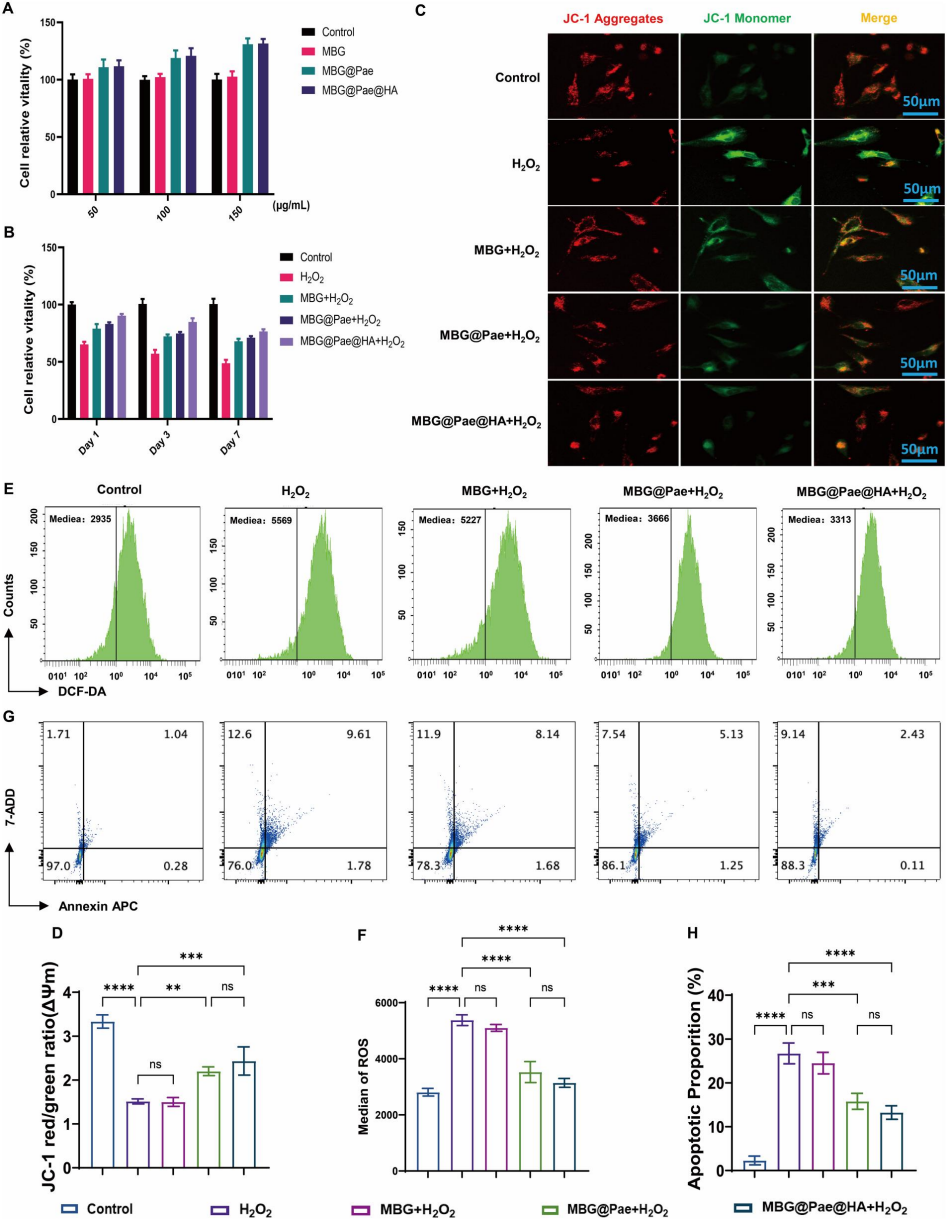
Protective and restorative effects of MBG@Pae@HA on chondrocyte function under oxidative stress. (**A**) Cytotoxicity of chondrocytes with or without MBG@Pae@HA treatment and (**B**) cytoprotection in H_2_O_2_-induced chondrocytes. (**C**) JC-1 staining was applied to visualize alterations in mitochondrial membrane potential of chondrocytes and (**D**) corresponding Statistical analysis. (**E**) Representative flow histograms using DCFH-DA as a fluorescent ROS probe and (**F**) the corresponding quantitative analysis of the intracellular ROS generation. (**G**, **H**) Representative flow cytometric scatter plot and quantification analysis of chondrocytes apoptosis after treatment with H_2_O_2_ and MBG@Pae@HA. (**P < 0.01, ***P < 0.001, ****P < 0.0001).

To verify potential biomedical applications, the cytotoxicity of MBG@Pae@HA was initially assessed in rat primary chondrocytes (Figure 4A). All the groups showed no cytotoxicity. Notably, the cell viability of MBG@Pae and MBG@Pae@HA groups was slightly higher than that of control and MBG. Moreover, the free Pae showed no cytotoxicity (Supplementary Figure S5). The protective effects of MBG@Pae@HA against H_2_O_2_-induced oxidative stress were also analysed by CCK8 assay (Figure 4B). While H_2_O_2_ treatment alone significantly reduced cell viability compared to the untreated control, MBG has no protective effect on cells damaged by H_2_O_2_. Notably, MBG@Pae and MBG@Pae@HA exhibited enhanced cytoprotection. These findings highlight the significant increase in the cell viability of MBG achieved by Pae loading and HA modification.

Next, we assessed the mitochondrial membrane potential (MMP), intracellular ROS level and apoptosis of cells in MBG@Pae@HA. The MMP of cells was quantitatively assessed using JC-1 fluorescence probe, which exhibits MMP-dependent accumulation in mitochondria. As shown in Figure 4C and D, H_2_O_2_ treatment alone caused severe mitochondrial depolarization, as evidenced by a reduction in red/green ratio compared to control group. The addition of MBG showed no difference in the MMP after H_2_O_2_ damage. Most remarkably, MBG@Pae and MBG@Pae@HA exhibited increased MMP compared to H_2_O_2_ and MBG+H_2_O_2_, demonstrating the Pae’s antioxidant properties. With the addition of H_2_O_2_, the ATP yield and GSH level of the chondrocytes were largely suppressed, while the application of MBG@Pae and MBG@Pae@HA revived the mitochondrial function of ATP production and GSH to the level of normal chondrocytes (Supplementary Figures S6 and S7). The ROS generation level in Figure 4E and F demonstrated the antioxidant efficacy of different nanoparticles against H_2_O_2_-induced intracellular oxidative stress. The cells in control group maintained basal ROS levels, while H_2_O_2_ treatment triggered increase in ROS production. The addition of MBG did not significantly alter the ROS levels induced by H_2_O_2_ exposure. The MBG@Pae+H_2_O_2_ and MBG@Pae@HA+H_2_O_2_ combination exhibited enhanced protection, decreasing ROS levels compared to control. These results suggest that Pae loading contributes additional antioxidant activity. The complete ROS normalization by MBG@Pae@HA highlights its potential for oxidative stress management in inflammatory conditions.

Additionally, we assessed the protective effect of MBG, MBG@Pae and MBG@Pae@HA with chondrocytes against H_2_O_2_-induced programmed cell death (Figure 4G and H). Our findings revealed that H_2_O_2_ treatment and MBG+H_2_O_2_ significantly increased apoptosis. MBG@Pae and MBG@Pae@HA demonstrated enhanced anti-apoptotic properties efficacy against H_2_O_2_-induced cell apoptosis. Subsequently, cell viability was further assessed through live/dead double-fluorescence staining using a Calcein-AM/PI assay kit [37]. H_2_O_2_ caused a large amount of cell death, and the situation was alleviated with the protection of the MBG@Pae and MBG@Pae@HA (Supplementary Figures S8 and S9). As a crucial enzyme in glycolysis, LDH leaks into extracellular space when plasma membrane integrity is compromised. Treatment with MBG@Pae or MBG@Pae@HA effectively suppressed the elevation of LDH levels triggered by H_2_O_2_ (Supplementary Figure S10). These results indicate that the MBG@Pae and MBG@Pae@HA can significantly protect chondrocytes from damage by excessive ROS.

As essential antioxidant enzymes, SOD and CAT function as endogenous scavengers of superoxide anions and hydrogen peroxide, respectively, thereby protecting the organism from oxidative stress-induced damage. MBG@Pae and MBG@Pae@HA rescued the SOD activity (Supplementary Figure S11) and CAT activity (Supplementary Figure S12) level of H_2_O_2_-induced chondrocytes, thereby inferring a well-restored ROS-regulating function in chondrocytes. Malondialdehyde (MDA) is an indicator of ROS-induced lipid peroxidation. A lipid peroxidation (MDA) assay kit proved that MBG@Pae and MBG@Pae@HA notably downregulated the elevated MDA level induced by H_2_O_2_ (Supplementary Figure S13). Therefore, MBG@Pae and MBG@Pae@HA could effectively eliminate intracellular ROS.

Experimental evidence demonstrates Pae’s ability to decrease oxidative stress markers (e.g. MDA) while upregulating key antioxidant enzymes (SOD, CAT), which are also reported in the literature [38, 39]. The compound exerts these effects primarily through activation of the Nrf2/HO-1 pathway, a master regulator of cellular redox balance [40]. Importantly, Pae protects chondrocytes from ROS-mediated apoptosis and mitochondrial dysfunction—pathological hallmarks of osteoarthritis [38]. In OA rat models, Pae attenuated cartilage degeneration by simultaneously reducing oxidative stress and suppressing NF-κB-driven inflammation [12], while in bone tissue it counteracted H_2_O_2_-induced osteoblast impairment by maintaining mitochondrial function and enhancing differentiation capacity [41]. Collectively, these findings establish Pae as a promising multi-target therapeutic agent for oxidative stress-related musculoskeletal disorders by restoring cellular ROS homeostasis and attenuating downstream chondrocyte inflammation.

### Restoring chondrocyte function and enhancing BMSC osteogenesis under oxidative stress via MBG@Pae@HA

**Figure 5:**
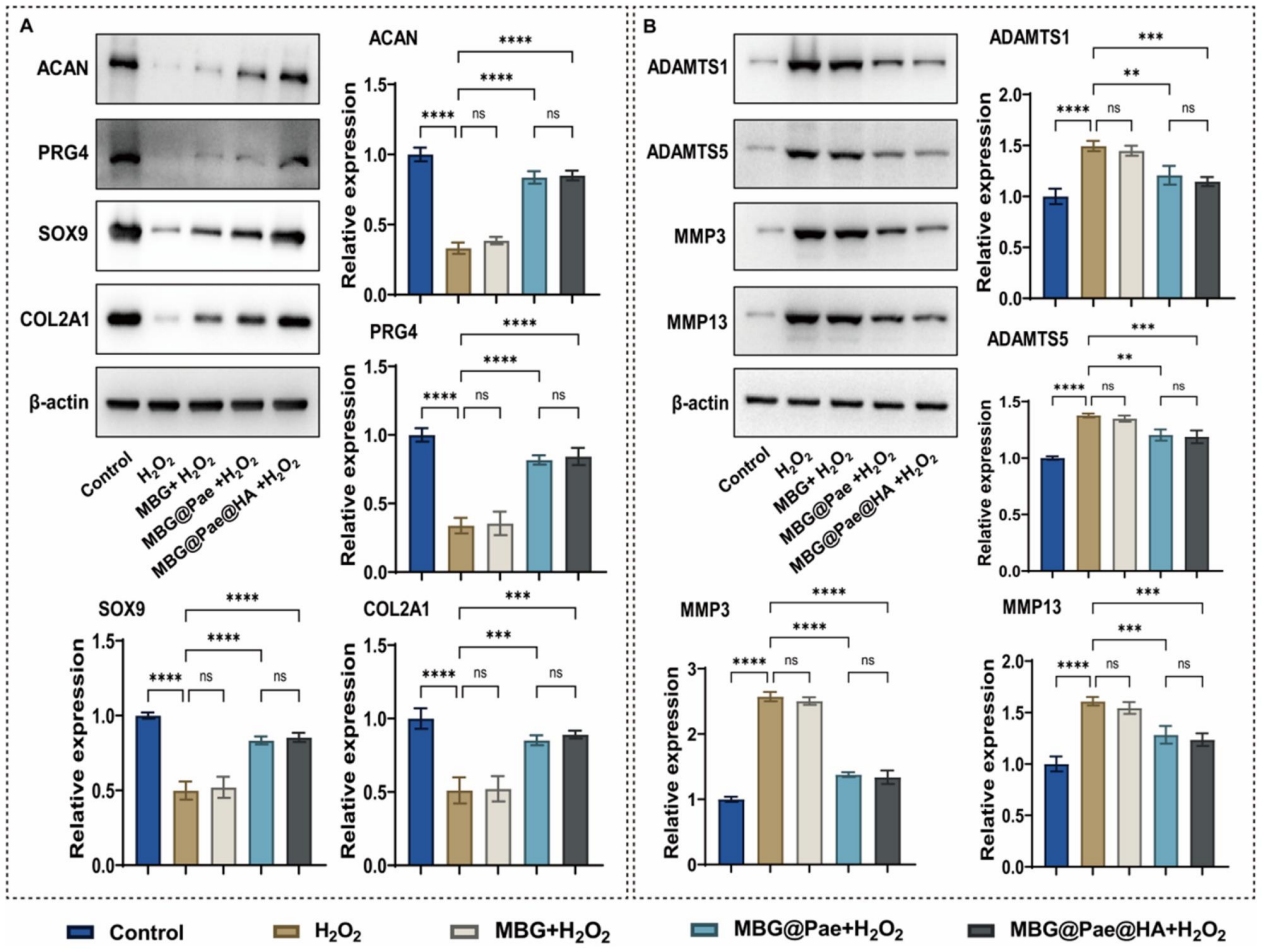
Western blot and RT-qPCR analysis of (**A**) ACAN, PRG4, SOX9 and COL2A1, as well as (**B**) ADMMTS1, ADMMTS5, MMP3 and MMP13 expression in chondrocytes. (**P < 0.01, ***P < 0.001, ****P < 0.0001).

Beyond energy metabolism, cartilage homeostasis critically depends on the balance between matrix anabolism and catabolism. The articular hyaline cartilage matrix, predominantly composed of type II collagen (COL2A1), aggrecan (ACAN) and proteoglycan 4 (PRG4), plays a critical role in maintaining proper physiological functions of load-bearing and lubrication in the joint [42, 43]. SRY-box transcription factor 9 (SOX9) has been demonstrated to play a pivotal role in chondrogenesis by promoting COL2A1 expression [44]. Accordingly, Western blotting and RT-qPCR analyses of these proteins and genes were conducted (Figure 5A and Supplementary Figure S14). The impaired expression of COL2A1, ACAN, PRG4 and SOX9 in H_2_O_2_-induced chondrocytes was markedly restored in the MBG@Pae- and MBG@Pae@HA-treated groups, indicating the remarkable protective effects of MBG@Pae and MBG@Pae@HA against inflammation- and ROS-induced chondrocyte dysfunction in OA.

ADAMTSs (a disintegrin and metalloproteinase with thrombospondin motifs, e.g. ADAMTS5, ADAMTS1) and MMPs (matrix metalloproteinases, e.g. MMP3 and MMP13) play pivotal roles in cartilage matrix degradation during OA, contributing to cartilage erosion and subchondral bone remodeling [8, 45, 46]. Herein, the expression of these proteins and genes was subsequently detected (Figure 5B and Supplementary Figure S14). H_2_O_2_ stimulation markedly activated cartilage catabolism and induced the upregulation of ADAMTS5, ADAMTS1, MMP3 and MMP13 in chondrocytes. Treatment with MBG@Pae and MBG@Pae@HA effectively counteracted this upregulation, indicating their potent inhibitory effects on activated catabolic processes in OA chondrocytes. Overall, MBG@Pae and MBG@Pae@HA treatment significantly improved the metabolic activity of chondrocytes and suppressed cartilage degradation in OA. Moreover, immunofluorescence (IF) analyses show enhanced COL2A1 deposition and suppressed MMP13 expression in the MBG@Pae@HA group compared with the other groups, supporting a preserved chondrocyte phenotype at the protein level (Supplementary Figure S15).

**Figure 6:**
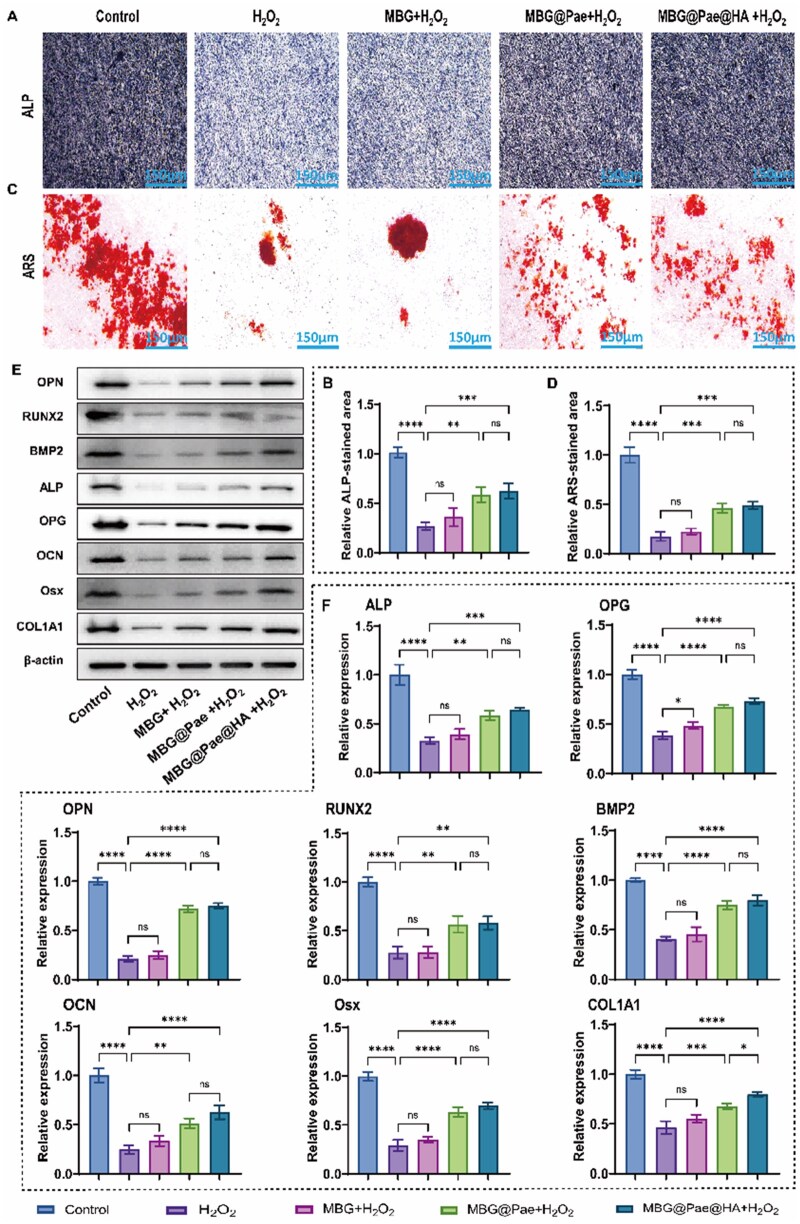
MBG@Pae@HA facilitate osteogenic differentiation of BMSCs. (**A**) ALP staining illustrating osteogenic activity across different treatment groups. (**B**) Comparative analysis of ALP enzymatic activity in BMSCs subjected to the indicated treatments. (**C**) ARS staining (**D**) quantitative analysis of calcium nodule under different treatments. (**E**, **F**) Western blot analysis and RT-qPCR analysis of bone-related proteins and genes in BMSCs, respectively. (**P < 0.01, ***P < 0.001, ****P < 0.0001).

In addition to its effects on chondrocytes, MBG@Pae@HA also modulates the osteogenic activity of BMSCs. ALP staining revealed that oxidative stress can inhibit the expression of ALP in BMSCs, whereas more ALP activity was noted in the MBG@Pae+H_2_O_2_ and MBG@Pae@HA+H_2_O_2_ groups over the other H_2_O_2_ damaged groups (Figure 6A). Figure 6B demonstrated that the relative ALP-stained area was large in MBG@Pae+H_2_O_2_ and MBG@Pae@HA+H_2_O_2_ groups over the other H_2_O_2_ damaged groups (H_2_O_2_ and MBG+H_2_O_2_ group). Alizarin red staining (ARS) was performed on BMSCs cultured under oxidative stress for 21 days, with or without MBG@Pae@HA, to evaluate osteogenic mineralization (Figure 6C and D). BMSCs exposed to MBG@Pae or MBG@Pae@HA exhibited denser calcium nodule formation, reflecting improved osteogenic potential under *in vitro* oxidative stress. MBG@Pae and MBG@Pae@HA groups showed consistent upregulation of core osteogenic genes and proteins (Figure 6E and F and Supplementary Figure S16). In detail, early commitment-matrix maturation (RUNX-2/BMP-2/COL1A1/ALP/Osx) and late mineralization-matrix remodelling (OCN/OPN/OPG) collectively indicated a sustained enhancement of osteogenesis in these groups, supporting the beneficial effect of Pae-loaded MBG on subchondral bone repair under oxidative stress *in vitro*.

The regulation of osteogenic and chondrogenic behaviors by bioactive ions and phytochemicals such as Pae has emerged as a promising strategy for bone and cartilage regeneration [47]. For instance, Ca^2+^ has been shown to enhance osteogenic differentiation by activating the Wnt/β-catenin pathway while simultaneously suppressing osteoclast activity, thereby promoting a favorable bone-remodeling balance [48]. In cartilage tissue, bioactive ions such as Si^4+^ exhibit chondroprotective effects by reducing oxidative stress and inhibiting inflammatory cytokine production [49]. In addition, studies indicate that Pae promotes osteoblast differentiation, leading to increased ALP activity and calcium nodule formation [50]. Furthermore, in chondrocytes, Pae exerts anti-inflammatory and ECM-protective effects by inhibiting ferroptosis through p53/SLC7A11/GPX4 pathway, thereby reducing MMP-13 expression and preventing type II collagen degradation [51]. In this study, we utilized the antioxidant properties of Pae to protect chondrocytes from oxidative stress damage and promote subchondral bone formation in synergy with HA and bioactive ions. Future studies should explore combinatorial approaches to maximize therapeutic efficacy in bone and cartilage regeneration.

### Transcriptomic analysis of MBG@Pae@HA against H_2_O_2_-induced OA chondrocytes

**Figure 7:**
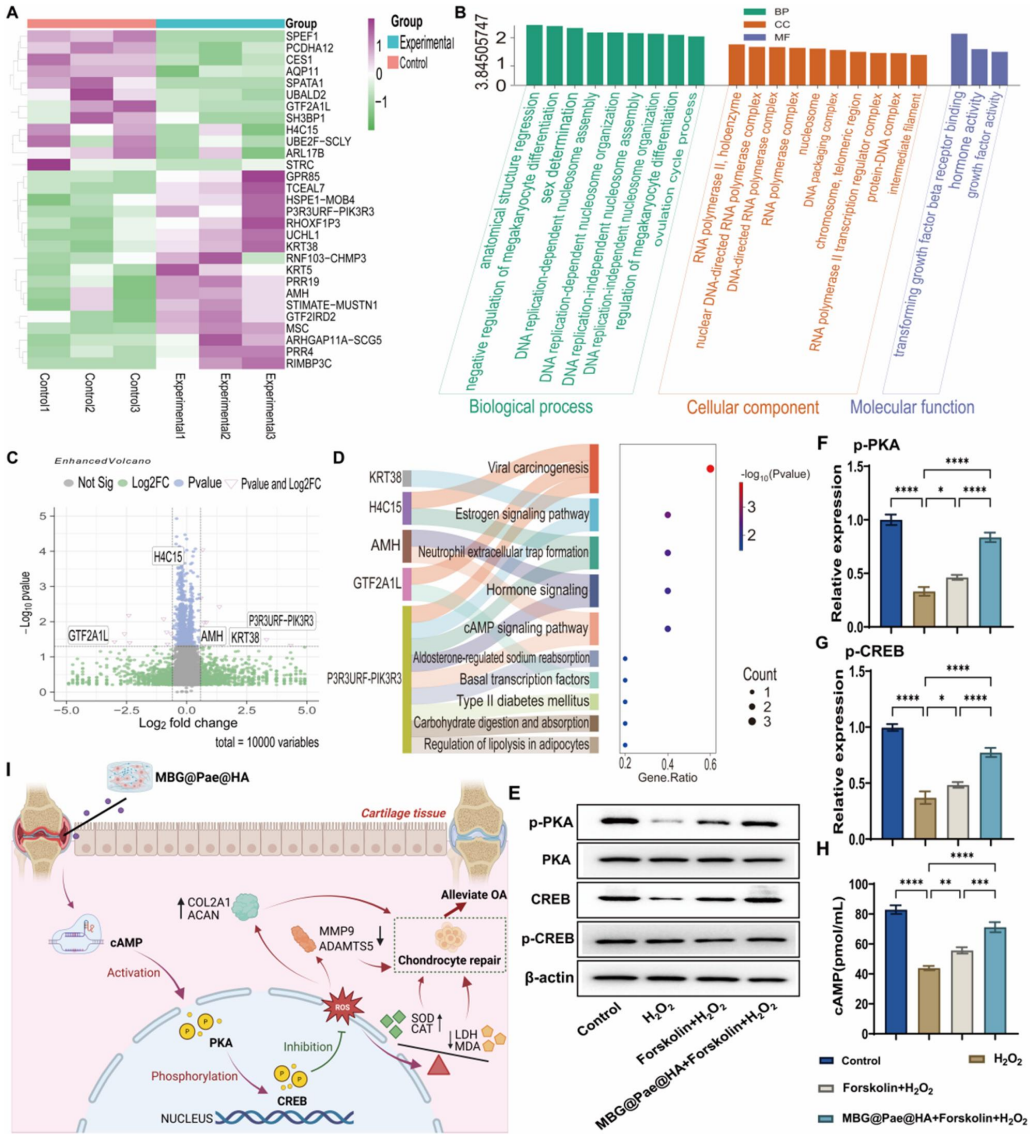
(**A**) Heatmaps illustrating sample clustering from pairwise comparisons. (**B**) Bar chart displaying GO enrichment analysis. (**C**) Volcano plots from pairwise comparisons. (**D**) Sankey diagram representing KEGG enrichment pathways. (**E**) Protein bands of p-PKA, PKA, CREB and p-CREB. (**F**) Relative expression levels of phosphorylated PKA protein. (**G**) Relative expression levels of phosphorylated CREB protein. (**H**) ELISA results for cAMP protein content. (**I**) Schematic mechanism illustrating how MBG@Pae@HA regulates the cAMP/PKA/CREB signaling pathway to exert therapeutic effects on OA. (*P < 0.05, **P < 0.01, ***P < 0.001, ****P < 0.0001).

Encouraged by the aforementioned findings, RNA sequencing was performed on H2O2-induced OA chondrocytes to further identify potential therapeutic targets affected by MBG@Pae@HA treatment. Initial target identification for MBG@Pae@HA-mediated OA chondrocyte improvement was performed following data background correction and normalization. Sample distribution and principal component analysis (PCA) plots are presented in Supplementary Figures S17 and S18, demonstrating high inter-group homogeneity, ensuring the reliability of subsequent analyses. Differential expression analysis, using thresholds of *P* values < 0.05 and a minimum 1.50-fold change (|log_2_FC| ≥ 0.58), identified 17 significantly upregulated and 12 downregulated genes in MBG@Pae@HA-treated OA chondrocytes. The corresponding heatmap and volcano plot visualizing these differentially expressed genes (DEGs) are shown in Figure 7A and C.

The expression matrix of DEGs was subjected to weighted gene co-expression network analysis (WGCNA). A soft-thresholding power (β) of 4 was selected when the scale-free topology fit index first exceeded 0.9 (Supplementary Figure S19), indicating suitability for network construction. Sample clustering analysis based on expression profiles of all six samples revealed no outliers (Supplementary Figure S20), confirming data quality for subsequent analyses. Using *β *= 4, we constructed the co-expression network and performed hierarchical clustering with a topological overlap matrix. Dynamic tree cutting identified 20 distinct gene modules (Supplementary Figure S21). Module-trait correlation analysis demonstrated that the MEdarkturquoise module showed the strongest association with MBG@Pae@HA-mediated OA chondrocyte improvement (|r|≥0.80, *P* < 0.05; Supplementary Figure S22), suggesting its potential as a hub module containing 88 key genes. Intersection analysis between the 29 DEGs and 88 hub genes identified five potential therapeutic targets (H4C15, GTF2A1L, P3R3URF-PIK3R3, AMH and KRT38; Supplementary Figure S23). Finally, expression patterns of WGCNA-identified genes across clusters were visualized in a heatmap (Supplementary Figure S24).

To further elucidate the key biological processes and signaling pathways influenced by MBG@Pae@HA in OA chondrocytes, Gene Ontology (GO) and Kyoto Encyclopedia of Genes and Genomes (KEGG) pathway enrichment analyses were conducted. The results demonstrated significant enrichment in key biological processes and signaling pathways, including DNA-templated transcription initiation, estrogen signaling pathway, neutrophil extracellular trap formation, hormone signaling and cAMP signaling pathway (Figure 7B and D). GO and KEGG enrichment analyses identified the cAMP signaling pathway as the predominant pathway mediating MBG@Pae@HA’s therapeutic effects in H_2_O_2_-induced OA chondrocytes. Forskolin was selected as the positive control drug, which directly activates adenylyl cyclase to promote the conversion of ATP to cAMP, thereby initiating the downstream cAMP-dependent signaling pathway. Experimental validation demonstrated that MBG@Pae@HA treatment significantly upregulated cAMP levels and enhanced p-PKA, PKA, p-CREB and CREB expression in H_2_O_2_-induced OA chondrocytes (Figure 7E–H).

It is noteworthy that PKA phosphorylates the transcription factor CREB (cAMP response element-binding protein), thereby enhancing the expression of downstream target genes (e.g. c-Fos) and regulating cellular metabolism, proliferation and antioxidant defense. Notably, this pathway is redox-sensitive. The PKA regulatory subunit RI can be oxidatively activated via disulfide bond formation at cysteine residues (Cys17/Cys38), independent of cAMP signaling, thereby modulating chondrocyte function under pathological conditions. For instance, activation of the cAMP/PKA/CREB axis significantly upregulates SOX9 expression, promoting the synthesis of cartilage-specific extracellular matrix components, including type II collagen and aggrecan. Furthermore, this pathway enhances the expression of antioxidant enzymes such as SOD2 and catalase, mitigating oxidative stress. A recent study developed the stimuli-responsive cAMP-activating drug delivery systems that selectively target damaged cartilage, effectively reducing intra-articular ROS levels and delaying OA progression [52, 53]. Therefore, a schematic illustration of this mechanistic pathway is presented in Figure 7I. MBG@Pae@HA releases activate the cAMP/PKA/CREB signaling cascade, leading to enhanced PKA phosphorylation and subsequent CREB nuclear translocation. These molecular events collectively ameliorate oxidative stress through modulation of SOD/CAT and correct metabolic dysfunction by regulating LDH/MDA balance, ultimately resulting in chondroprotective effects and significant alleviation of osteoarthritic pathology.

### *In vivo* treatment efficacy of MBG@Pae@HA for OA

**Figure 8:**
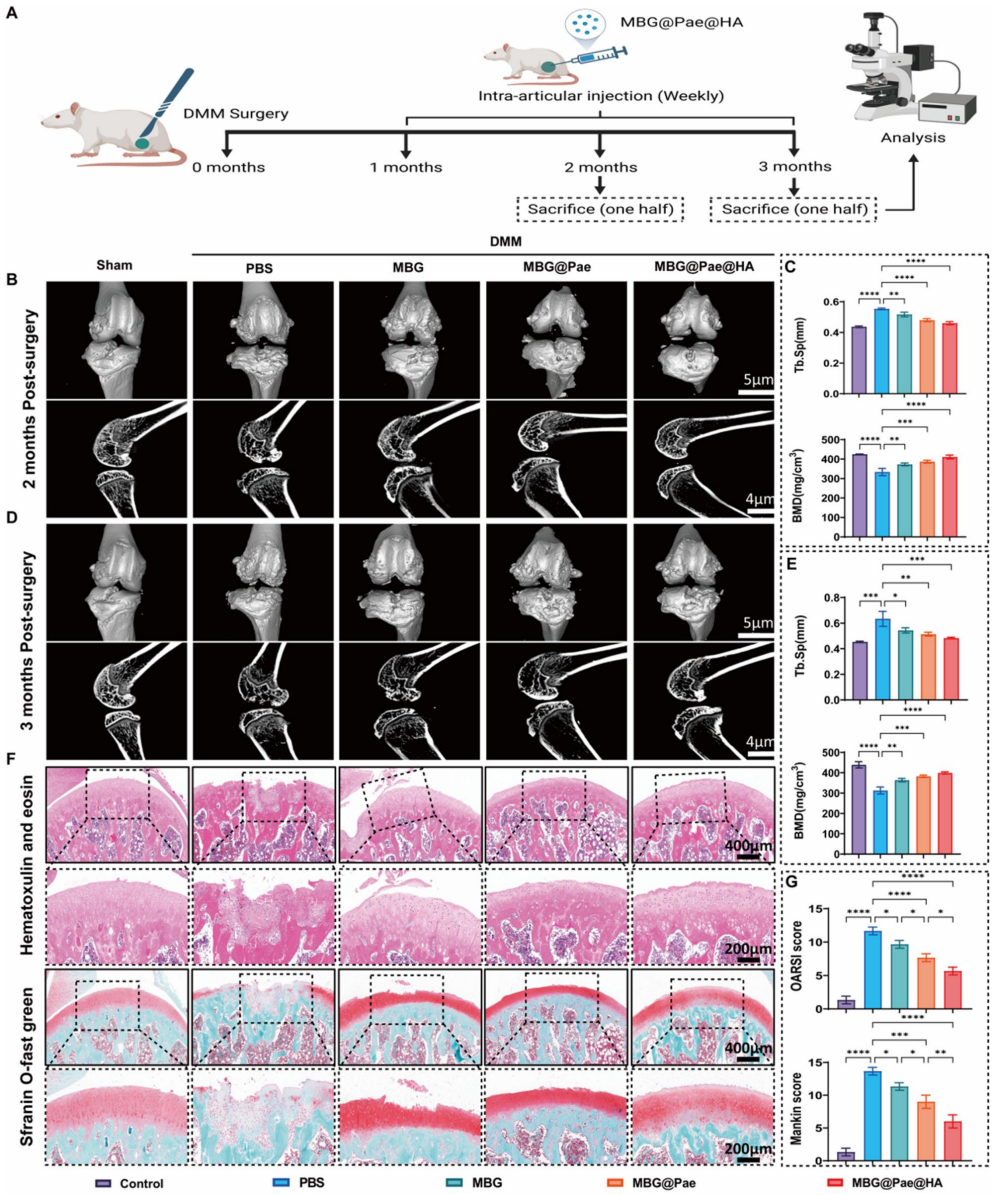
MBG@Pae@HA attenuated OA progression *in vivo*. (**A**) Schematic depiction outlining the time points for rats undergoing sham or DMM surgery, intervention and imaging. PBS, MBG, MBG@Pae and MBG@Pae@HA were intra-articular injected once a week, continuous injection for 4 weeks and rats were sacrificed on 2 months and 3 months for analysis. (**B**–**E**) Micro-CT imaging of knee joints and quantitative assessment of BMD and Tb. Sp after 2 and 3 months of treatment. (**F**) Representative histological sections stained with H&E and so-FG and (**G**) evaluation of OARSI and Mankin scores at 2 months post-surgery in various treatment groups. (*P < 0.05, **P < 0.01, ***P < 0.001, ****P < 0.0001).

The *in vivo* efficacy of MBG@Pae@HA in OA was evaluated using a rat knee OA model induced by medial meniscus destabilization (DMM). MBG@Pae@HA (5 mg/kg, 10 µL) was administered via intra-articular injection starting 2 weeks post-operation, at a frequency of once every 7 days for a total of 12 weeks (Figure 8A). Control rats subjected to DMM surgery to induce OA and sham-operated rats serving as normal controls were both given normal saline at the corresponding time points. Owing to the excellent ROS-scavenging capacity and bioactive ions (Si, Ca, ${\text{PO}}_{4}^{3-}$), MBG@Pae@HA achieves optimal therapeutic efficacy at a low dosage. Furthermore, the intra-articular injection instead of intravenous injection prevents systemic phosphorus release, ensuring enhanced biosafety.

In DMM-operated control rats, 3D reconstructions revealed typical OA features such as osteophytes and joint deformity, whereas joints in MBG@Pae@HA-treated rats remained largely intact (Figure 8B and D). Moreover, analysis of coronal micro-CT sections of the medial tibial plateau revealed distinct subchondral bone changes among groups, with control OA rats showing marked bone loss and reduced trabecular density (Figure 7B and D, lower row). Quantitative micro-CT analysis revealed that the decreased trabecular bone mineral density (BMD), whereas increased trabecular separation (Tb. Sp), trabecular number (Tb.N) and bone volume fraction (BV/TV) observed in control OA knees were notably restored in the MBG@Pae@HA-treated groups (Figure 8C and E; Supplementary Figures S25 and S26).

Hematoxylin and eosin (H&E) and safranin-O/fast green (SO-FG) staining were subsequently performed after 2 months of treatment (Figure 8F and Supplementary Figure S27). Glycosaminoglycan and proteoglycan contents were markedly decreased in the control group, as evidenced by reduced TB and SO-FG staining, whereas these levels were restored following MBG@Pae@HA treatment. Furthermore, the results of toluidine blue (TB) staining consistently corroborate the findings from H&E and Safranin O–Fast Green staining, showing enhanced chondroitin sulfate-rich matrix deposition in the treated groups (Supplementary Figure S28). Severe cartilage degeneration and subchondral bone damage were evident in control OA knees, whereas MBG@Pae@HA-treated groups maintained the integrity of both cartilage surface and subchondral bone. The protective effects of MBG@Pae@HA against OA were further confirmed by OA Research Society International (OARSI) and Mankin scores (Figure 8G and Supplementary Figure S29).

**Figure 9:**
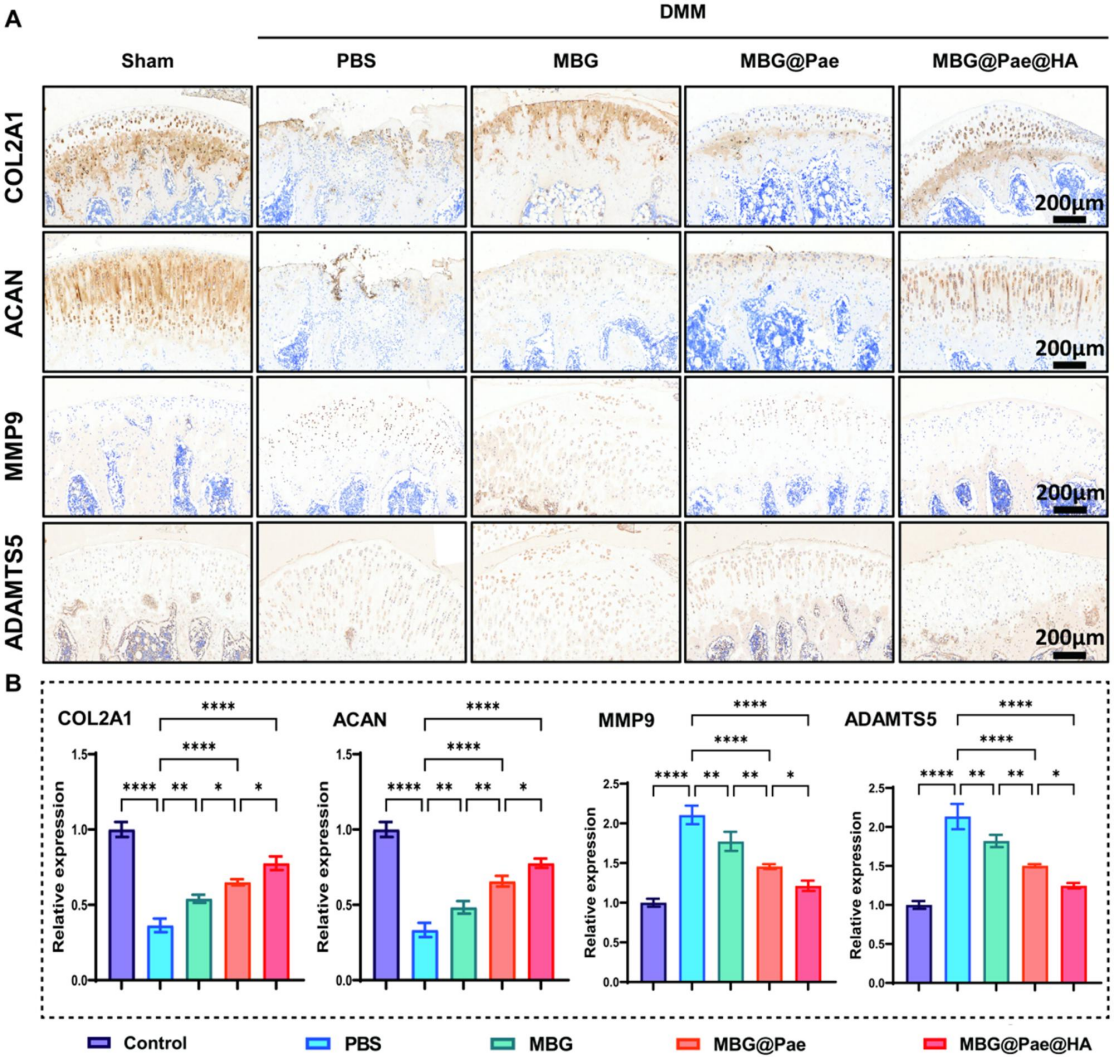
(**A**) Representative images of COL2A1, ACAN, MMP9 and ADAMTS5 immunohistochemistry staining of differently treated rat knees after 2 months of treatment. (**B**) Quantitative analysis of COL2A1, ACAN, MMP9 and ADAMTS5 expression. (*P < 0.05, **P < 0.01, ***P < 0.001, ****P < 0.0001).

Furthermore, the *in vivo* effects of MBG@Pae@HA were validated by immunohistochemical staining and quantification of osteogenesis-related proteins (COL2A1, ACAN, MMP9 and ADAMTS5) (Figure 9A and B and Supplementary Figures S30 and S31). The application of MBG@Pae@HA significantly enhanced cartilage biosynthesis while suppressing catabolic processes, as evidenced by upregulated COL2A1 and ACAN and downregulated MMP9 and ADAMTS5.

The well-documented biocompatibility of MBG in prior studies [54–56], which further supports the clinical application potential of MBG@Pae@HA. The absence of significant biotoxicity at the tested doses further highlights its translational potential in OA treatment, which also constitutes one of the key reasons for initially selecting MBG@Pae@HA as the nanomedicine. Overall, MBG@Pae@HA administered at the tested doses showed no apparent biotoxicity, highlighting their potential for clinical translation in OA therapy.

## Conclusion

To sum up, a novel nanogel composed of MBG, Pae and HA is constructed to treat OA. The MBG@Pae@HA nanogel shows enhance adhesion force to the tissue, and thus, offering an adaptive Pae and bioactive ions release at the OA lesion site. MBG@Pae@HA efficiently scavenged ROS generated in OA chondrocytes, thereby protecting chondrocyte biological functions against ROS-induced cartilage degradation via activation of the cAMP/PKA/CREB signaling pathway. Importantly, the released biodegradation products (Si^4+^, Ca^2+^, ${\text{PO}}_{4}^{3-}$) exhibited satisfactory *in situ* biomineralization and enhanced osteogenic differentiation of BMSCs, facilitating subchondral bone repair in OA. In an OA rat model, MBG@Pae@HA effectively preserved cartilage morphology and subchondral bone volume. With its excellent cartilage-protective and subchondral bone-repair capabilities, the MBG@Pae@HA nanogel shows promising potential for OA therapy.

## Experimental section

### Materials and reagents

All reagents were commercially obtained and used without further purification. CTAB, TEA, TEOS, cyclohexane, TEP, CNTH and HA were sourced from Macklin Biochemical Technology Co., Ltd. (Shanghai, China). Pae was acquired from Sigma-Aldrich. EDC, APTES and N-hydroxysuccinimide were purchased from Aladdin Co., Ltd. (Shanghai, China). Cy5.5, SF488 TUNEL kit (T2196), Safranine O Cartilage Stain Solution (G2540) and Calcein-AM/PI were obtained from Beijing Solarbio Science & Technology Co., Ltd.

### Synthesis of radial mesoporous bioglass microspheres (MBG)

The aqueous phase was first prepared by dissolving 12 g cetyltrimethylammonium bromide (CTAB, surfactant) and 0.36 mL triethanolamine (TEA, catalyst) in 108 mL deionized water within a 500 mL round-bottom flask under constant stirring (500 rpm) at 60°C in a thermostatic water bath. Simultaneously, the oil phase was formulated by mixing 12 mL tetraethyl orthosilicate (TEOS) with 48 mL cyclohexane under room temperature stirring. The oil phase solution was then added dropwise (0.5 mL/min) into the aqueous phase maintained at 60°C, with carefully controlled magnetic stirring to preserve distinct oil–water biphasic stratification throughout the 3 h reaction period. Following this, 0.921 mL triethyl phosphate (TEP) was introduced and the reaction continued for additional 9 h, yielding a milky white gel in the lower aqueous phase while maintaining a clear colorless upper oil phase. The product was isolated by phase separation, with the gel layer subsequently centrifuged and washed twice each with absolute ethanol and deionized water. The obtained white precipitate was redispersed in 20 mL absolute ethanol and reacted with calcium nitrate tetrahydrate solution (2.54 g in 20 mL ethanol) under 12 h stirring. After oven-drying at 60°C, the resultant white powder was heated in a muffle furnace at 600°C for 5 h to ultimately produce radiated mesoporous bioactive glass.

### Synthesis of MBG with paeoniflorin loading and hyaluronic acid modification (MBG@Pae@HA)

Initially, 2 g of MBG was mixed with 0.2 g of paeoniflorin (Pae) in absolute ethanol (50 mL) and stirred for 24 h to achieve drug loading. The resulting MBG@Pae was washed and dried. For surface amination, 1 g of MBG@Pae was dispersed in 100 mL of absolute ethanol, and then, reacted with 10 mL of APTES under continuous stirring at room temperature for 24 h. The aminated product (NH_2_-MBG@Pae) was collected via centrifugation, washed three times with absolute ethanol and redispersed in ultrapure water. Meanwhile, 50 mL of HA aqueous solution (10 mg/mL, pH 5.5–6.0) was activated by adding 287.5 mg of EDC and 172.5 mg of NHS, followed by shaking for 2 h. The NH_2_-MBG@Pae dispersion was then added to the activated HA solution and allowed to react for 3 h. The final MBG@Pae@HA conjugate was obtained after centrifugation and multiple washes to remove residual reagents.

### Material characterization

Morphological features, particle size distribution and surface charge of MBG@Pae@HA were examined by SEM, TEM and dynamic light scattering (DLS) with zeta potential analysis. The crystalline phase and chemical composition were determined through XRD, XPS, FTIR. TGA was conducted under dynamic air flow with a heating rate of 10°C/min from 30 to 700°C to quantify both the paeoniflorin loading capacity and HA hydrogel coating content.

### Adhesion and Young’s modulus determination

Atomic force microscopy (AFM) in contact mode (Cypher AFM, Asylum Research, Santa Barbara, CA, USA) was employed to evaluate the adhesion and Young’s modulus of MBG@Pae and MBG@Pae@HA. In brief, suspensions of MBG and MBG@Pae@HA were deposited onto freshly cleaved mica surfaces and allowed to air-dry. Force–indentation curves were recorded by positioning the AFM probe at the center of the particles and applying a load of 10 nN at a speed of 1 μm/s.

### Pae release test

The *in vitro* release profiles of MBG@Pae and MBG@Pae@HA were evaluated by dispersing 2 mg samples in 2 mL phosphate-buffered saline (PBS) followed by incubation in an orbital shaker (37°C, 60 rpm). At predetermined time intervals, the suspensions were centrifuged and the supernatants were collected for UV−Vis spectrophotometric analysis at *λ* = 230 nm. After each sampling, 2 mL fresh PBS was replenished to maintain sink conditions. The paeoniflorin concentration was quantified using a pre-established calibration curve (0–20 μg/mL) with seven concentration points (0, 0.5, 1, 2.5, 5, 10 and 20 μg/mL).

### Free-radical-scavenging capability of MBG@Pae@HA

The free-radical-scavenging capability of MBG@Pae@HA was evaluated using multiple assays. Typical antioxidant activity tests, including DPPH·, ABTS·+, ·$O_{2}^{-}$ and ·OH scavenging assays, were performed and monitored via UV−Vis spectroscopy.

#### Hydroxyl radical (·OH) clearance

OH Scavenging Capacity Assay Using Methylene Blue (MB): The **·**OH scavenging capacity was quantitatively evaluated through MB decolorization, where the residual MB absorbance reflects radical elimination efficiency. The Fenton reaction system was established by mixing 0.1 mL FeCl_2_·4H_2_O (5 mM), 0.1 mL H_2_O_2_(20 mM) and 1.7 mL deionized water. After 5 min incubation, 0.1 mL nanomaterial suspension (20 mg/mL) and 0.1 mL MB (1 mM) were sequentially added, followed by 3 h static reaction. Absorbance measurements at *λ* = 664 nm were performed for: pristine MB solution ($A_{m}$), MB+·OH mixture ($A_{n}$) and MB+**·**OH+nanomaterial ($A_{k}$). The scavenging efficiency (%) was calculated as:


$$\frac{A_{k}-A_{n}}{A_{m}-A_{n}}\times 100\%.$$

#### Superoxide anion (·$O_{2}^{-}$) clearance

Pyrogallol Autoxidation Assay was carried for the evaluation of superoxide anion (**·**$O_{2}^{-}$) scavenging capacity. The **·**$O_{2}^{-}$ scavenging activity was determined by monitoring pyrogallol autoxidation, which generates **·**$O_{2}^{-}$ and colored intermediates under weak alkaline conditions (pH 8.5), with absorbance intensity proportional to **·**$O_{2}^{-}$ concentration. Briefly, 0.1 mL nanomaterial suspension (20 mg/mL) was dispersed in 2 mL Tris-HCl buffer (50 mM, pH 8.5), followed by addition of 25 μL pyrogallol solution (25 mM). After 20 min incubation at 25°C, absorbance was measured at 319 nm for both control (pyrogallol only, A_Z_) and test (pyrogallol + nanomaterial, A_X_) samples. The **·**$O_{2}^{-}$ inhibition rate (%) was calculated as:


$$\cdot O_{2}^{-}\;\mathrm{inhibition}\;\mathrm{rate}\;(\%)=\frac{A_{z}-A_{x}}{A_{z}}\times 100\%.$$

#### DPPH ˙ assay

The stable free radical DPPH was utilized to assess the antioxidant activity. A 0.2 mg/mL DPPH solution was prepared by dissolving 4 mg DPPH in 20 mL absolute ethanol. For the assay, 0.1 mL of the sample suspension (20 mg/mL) was combined with 1.9 mL of DPPH solution and incubated in the dark at room temperature for 30 min. Absorbance was then recorded at 519 nm, and the radical scavenging efficiency (%) was calculated as follows:


$$\mathrm{DPPH}\cdot \;\mathrm{scavenging}\;\mathrm{rate}\;(\%)=\frac{A_{c}-A_{s}}{A_{c}}\times 100\%,$$

where *A_c_* represents the absorbance of the control (DPPH solution without sample) and *A_s_* denotes the absorbance of the test mixture.

#### In the ABTS^+.^ assay

The ABTS^+.^ radical cation was generated by reacting 0.4 mL ABTS [2,2'-azino-bis(3-ethylbenzothiazoline-6-sulfonic acid] diammonium salt, 7.4 mM, prepared by dissolving 4.5 mg in 1.1 mL deionized water, Mw 548.7) with 1.43 mL potassium persulfate [(NH_4_)_2_S_2_O_8_, 2.6 mM in deionized water], followed by overnight incubation in the dark at room temperature. The resulting ABTS^+.^ stock solution was diluted 20-fold with absolute ethanol to obtain the working solution.

For the assay, 0.1 mL of sample suspension (20 mg/mL in ethanol) was mixed with 1.9 mL ABTS^+^**·** working solution and incubated for 6 min under dark conditions. Absorbance was measured at 734 nm for:


$$\text{Control}\;\left(A_{0}\right):\;1.{9\;\text{mL}\;\text{ABTS}}^{+}\cdot \text{working}\;\text{solution}+0.1\;\text{mL}\;\text{absolute}\;\text{ethanol}\text{Test}\;\left(A\right):\;1.{9\;\text{mL}\;\text{ABTS}}^{+}\cdot \text{working}\;\text{solution}+0.1\;\text{mL}\;\text{sample}\;\text{suspension}$$

The radical scavenging activity was calculated as:


$$\mathrm{ABTS}+\cdot \mathrm{scavenging}\;\mathrm{rate}\;(\%)=\frac{A_{0}-A}{A_{0}}\times 100\%$$

### Cell culture

Primary chondrocytes and bone marrow mesenchymal stem cells (BMSCs) were isolated from 2-week-old Sprague–Dawley rats (Shanghai Slac Laboratory Animal Co., Ltd.). For chondrocyte isolation, femoral head cartilage was aseptically excised, cut into ∼1 mm³ pieces and sequentially digested with 0.25% trypsin at 37°C for 30 min, followed by 0.025% collagenase type II overnight. The harvested chondrocytes were cultured in high-glucose DMEM supplemented with 10% FBS and 1% penicillin/streptomycin. BMSCs were isolated by flushing the femurs and tibias with complete medium until marrow removal.

### Cytocompatibility investigation

Primary chondrocytes were seeded in 96-well plates and cultured overnight before treatment with MBG@Pae@HA. After 24 h incubation, cell viability was assessed using CCK-8 assay by replacing medium with 100 μL fresh medium containing 10 μL CCK-8 reagent, incubating for 2 h, then, adding 100 μL detergent reagent for 4 h dark incubation prior to measuring absorbance at 570 nm (Tecan microplate reader). This protocol was similarly applied for time-course studies and comparative evaluation of differently prepared MBG samples.

### Protective effect of MBG@Pae@HA on chondrocytes against ROS

Primary chondrocytes were seeded onto culture plates and treated with H_2_O_2_ (100 μM) or LPS (1 μg/mL) for 24 h to establish oxidative stress or OA cellular models. MBG@Pae@HA (100 μg/mL) was co-administered with H_2_O_2_ or LPS. Cell viability was evaluated using CCK-8 and Calcein-AM/PI live/dead double staining under 100 μM H_2_O_2_ treatment. Mitochondrial membrane potential (MMP) was assessed with a JC-1 assay kit (Servicebio, G1515) using a microplate reader and confocal laser scanning microscopy. Intracellular ROS levels were measured using the fluorescent probe DCFH-DA incubated for 30 min, followed by PBS washing. Fluorescence signals from live/dead and ROS staining were captured by fluorescence microscopy and quantified with Image J software. SOD activity in H_2_O_2_-induced chondrocytes was determined using a total SOD assay kit (Beyotime, S0101S) with WST-8. Lipid peroxidation was evaluated with an MDA assay kit (Beyotime, S0131S). ATP and CAT activities in H_2_O_2_ -treated cells were measured using ELISA kits (Beyotime, S0026 and Solarbio, BC0205, respectively). LDH and GSH levels were analyzed using LDH (Beyotime, C0016) and GSH (Solarbio, BC1175) assay kits.

### RNA sequencing of chondrocytes

Primary chondrocytes were seeded onto culture plates and assigned to two groups. The control group was treated with H_2_O_2_ (10 μM) to establish OA chondrocytes, with three replicates per condition. In the MBG@Pae@HA group, cells were exposed to H_2_O_2_ (100 μM) together with MBG@Pae@HA (100 μg/mL), also in triplicate. After 24 h of incubation, cells were harvested and total RNA was extracted using Trizol reagent. The RNA samples were subsequently subjected to sequencing on a BGI sequencing platform.

#### Data preprocessing and differential analysis

To elucidate the therapeutic targets of MBG@Pae@HA in OA chondrocytes, high-throughput transcriptome sequencing was performed on two groups of chondrocyte samples. Raw microarray data underwent background correction, normalization and expression quantification using Bioconductor packages in R. Differentially expressed genes (DEGs) were identified via the limma package, with screening thresholds set at adjusted *P* values <0.05 and |log_2_FC| >0.58, thereby pinpointing key DEGs modulated by MBG@Pae@HA in OA chondrocytes. The heatmap and volcano plot packages were employed to visualize clustered DEGs, with hierarchical analysis conducted to delineate distinct gene expression profiles.

#### Weighted gene co-expression network construction

Genes with expression variance above the upper quartile were selected for weighted gene co-expression network analysis (WGCNA) to investigate the effects of MBG@Pae@HA in OA chondrocytes. The analysis was performed using the R package “WGCNA.” Sample clustering was first conducted, and the optimal soft-thresholding power (β) was determined according to scale-free topology criteria. An adjacency matrix was generated via β-powered transformation, followed by calculation of the topological overlap matrix (TOM) to evaluate gene dissimilarity. Hierarchical clustering based on TOM dissimilarity was applied to identify modules through dynamic tree cutting, with closely related modules subsequently merged. Module eigengenes (MEs) were calculated to summarize the expression pattern of each module. Pearson correlation analysis between MEs and clinical traits was conducted to identify the module most associated with MBG@Pae@HA intervention. This key module was then selected for further gene screening and functional enrichment analysis.

#### Enrichment analysis

To systematically identify the key target proteins through which MBG@Pae@HA ameliorates OA chondrocytes, GO and KEGG enrichment analyses were performed using the ClusterProfiler R package from Bioconductor. The GO analysis encompassed cellular components (CC), molecular functions (MF) and biological processes (BP) to elucidate the functional attributes of the core targets. Enriched GO and KEGG terms were selected based on ascending *P*-values and visualized to highlight the most statistically significant pathways.

### *In vitro* osteogenic differentiation of BMSCs

BMSCs were cultured in osteogenic differentiation medium (DMEM supplemented with 10 mM β-sodium glycerophosphate, 50 μg/mL L-ascorbic acid and 10 nM dexamethasone) with or without MBG@Pae@HA intervention for 21 days. Calcium nodule formation was assessed by alizarin red S staining per manufacturer’s protocol. Osteogenic differentiation was further verified through RT-qPCR analysis of OPN, RUNX2 and BMP2 gene expression (primer sequences provided in Supplementary Table S1).

### Quantitative reverse transcription polymerase chain reaction

After chondrocyte treatment, cell samples were stabilized in RNAlater solution (Thermo-Fisher Scientific), followed by RNA extraction using TRIzol reagent (Takara, Japan). Complementary DNA (cDNA) was synthesized using the iScript cDNA synthesis kit (Bio-Rad, Hercules, CA). Gene expression levels were assessed via SYBR Green-based quantitative reverse transcription PCR (qRT-PCR), performed with Thermo-Fisher Scientific reagents (Waltham, MA) and gene-specific primers (listed below), adhering to the manufacturer’s protocol. Amplification specificity was confirmed by melt curve analysis and agarose gel electrophoresis, with each sample analyzed in triplicate. β-actin was used as the housekeeping gene. The relative primer sequences were listed in Supplementary Table S1. The relative expression was calculated according to the 2^−ΔΔCt^ method.

### Western blotting

After nanomaterials treatment, chondrocytes were trypsinized, pelleted by centrifugation and washed with PBS. Total protein was extracted using RIPA lysis buffer (PC102, Epizyme Biomedical Technology, Shanghai) for 30 min at 4°C. Following heat denaturation, proteins were separated by electrophoresis and transferred to a PVDF membrane. After 1 h of blocking with QuickBlock™ Western (P0252, Beyotime Biotechnology), the membrane was incubated with primary antibodies overnight at 4°C. The information of primary antibodies were showed in Supplementary Table S2. β-actin served as the loading control. A horseradish peroxidase (HRP)-conjugated secondary antibody (HA1001, HUABIO; 1:50 000) was used for detection. For cAMP quantification, a commercial ELISA kit (E-EL-0056, Elabscience) was employed. Forskolin (66575-29-9, Absin), a cAMP agonist, was used in functional assays.

### The DMM surgeries and intra-articular injections for rats

All experimental procedures were approved by the ethics committee of the Animal Ethics Committee of Yunnan University of Chinese Medicine (YNUTCM-XMSS-G-20250056). To establish a mechanical instability-induced OA model, we surgically destabilized the medial meniscotibial ligament (DMM) in the right knee joints of 8-week-old male rats. OA progression was confirmed 4 weeks postoperatively, while control rats received sham surgery. Therapeutic interventions commenced at 1 month post-DMM surgery. Animals received weekly intra-articular injections (5 mg NPs/kg in 10 µl volume) for four consecutive weeks. For longitudinal analysis, knee joints were harvested 4 weeks and 8 weeks postsurgery. Rats were randomized into sham or DMM groups and administered weekly intra-articular injections (10 µl) of PBS (vehicle control), MBG, MBG@Pae or MBG@Pae@HA (5 mg NPs/kg) via subpatellar needle insertion. Body weight was monitored weekly to assess systemic health. *In vivo* micro-CT imaging was performed at 1 and 3 months post-treatment. At the endpoint, half of the rats were sacrificed for comprehensive analysis including Micro-CT, hematoxylin & eosin (H&E) and safranin O-fast green staining and immunohistochemical detection of COL2A1, ACAN, MMP9 and ADAMTS5.

### Micro-CT analysis

Dissected knee joints were fixed in 4% paraformaldehyde for 3 days. Following fixation, the knees were scanned using a micro-CT system (Nemo NMC-100, PINGSENG Healthcare Inc., China). The obtained imaging data were analyzed with Avatar software 1.6.6 to generate three-dimensional reconstructions of the knee joints and to quantify trabecular bone parameters, including bone mineral density (BMD) and trabecular separation (Tb. Sp).

### Statistical analysis

All data were analyzed using GraphPad Prism software. Results are expressed as mean ± standard deviation (SD). For comparisons involving more than two groups, multifactorial analysis of variance (ANOVA) was performed to evaluate statistical significance. *P < 0.05, **P < 0.01 and ***P < 0.001 were considered to be significant.

## Supplementary Material

rbag016_Supplementary_Data
