# Supplementary material for: Paeoniflorin-loaded MBG nanogel alleviates oxidative microenvironment and reinforces subchondral bone regeneration for osteoarthritis treatment
Source: Regen Biomater. 2026 Feb 5;13:rbag016. doi: 10.1093/rb/rbag016 (PMC13106897; doi:10.1093/rb/rbag016)
Supplement: rbag016_Supplementary_Data [file rbag016_supplementary_data.docx]

**Supporting Information**

**Paeoniflorin-loaded MBG Nanogel Alleviates Oxidative Microenvironment and Reinforces Subchondral Bone Regeneration for Osteoarthritis Treatment**

Zugui Wu^a,b,1^, Xiuhong Huang^c,1^, Jiao Li^a,1^, Gaoquan Zheng^d^, Feng Peng^d^, Wei Dong^a^, Yue Zhu^a^, Yu Zhang^d^, Rong Yuan^a^, Zhiwei Wu^a^, Ying Guo^a,^*, Jin Xiao^d,^*, Biaolin Wan^e,^*

*^a^The Third Clinical College of Yunnan University of Chinese Medicine, The Third Affiliated Hospital of Yunnan University of Chinese Medicine, Kunming, 650500, China*

*^b^Guangdong Cardiovascular Institute, Guangdong Provincial People’s Hospital (Guangdong Academy of Medical Sciences), Southern Medical University, Guangzhou, 510080, China*

*^c^School of Basic Medical Sciences, Guangzhou University of Chinese Medicine, Guangzhou 510006, China*

*^d^Medical Research Institute, Department of Orthopaedics, Guangdong Provincial People’s Hospital (Guangdong Academy of Medical Sciences), Southern Medical University, Guangzhou 510080, China*

*^e^Department of Joint Surgery and Sports Medicine, Heyuan People's Hospital, Guangdong Provincial People's Hospital Heyuan Hospital, Heyuan 517100, China*

**^*^ Corresponding Authors:**

E-mail addresses: gy1200@126.com (Y. Guo), xiaojin@gdph.org.cn (J. Xiao), [wanbiaolin4563743@163.com](mailto:wanbiaolin4563743@163.com) (B. Wan)
**^1^These authors contributed equally to this work**

**Table S1. The relative primer sequences utilized for qRT-qPCR**

| Target DNA | Forward sequence (5’- 3’) | Reverse sequence (3’- 5’) |
| --- | --- | --- |
| OPN | CACTCCAATCGTCCCTAC | AGACTCACCGCTCTTCAT |
| RUNX2 | ACACCGTGTCAGCAAAGC | GCTCACGTCGCTCATCTTG |
| BMP2 | GCCAAACACAAACAGCGGAA | CCAGTCATTCCACCCCACAT |
| ACAN | TTGTCTGAATGGAGCCACCT | ACACAGGTTCCCTCTGTAGC |
| PRG4 | CAAGAAGCCCACCTCTACCA | CACCTCCATCTGCATCTTCA |
| SOX9 | CGTGCAGCACAAGAAAGACCA | GCAGCGCCTTGAAGATAGCAT |
| COL2A1 | GCTGGTGAAGAAGGCAAACGAG | CCATCTTGACCTGGGAATCCAC |
| ADAMTS1 | GGCAAACGAGTCCGCTAC | CCCACTCTTCAATCACCCAC |
| ADAMTS5 | CCCAGGATAAAACCAGGCAG | CGGCCAAGGGTTGTAAATGG |
| MMP3 | GACGATGATGAACGATGGACAGAGG | TGTGGAGGACTTGTAGACTGGGTAC |
| MMP13 | TGTTTGCAGAGCACTACTTGAA | CAGTCACCTCTAAGCCAAAGAAA |
| ALP | CCTTCTTCCGTCAGTACCGT | AGCTGGTTCATCCCGATTGT |
| OPG | ACCCAGAAACTGGTCATCAGC | CTGCAATACACACACTCATCACT |
| OCN | CCTCTCTCTGCTCACTCTGCTG | CTATTCACCACCTTACTGCCCTC |
| Osx | AGCGACCACTTGAGCAAACA | GCGGCTGATTGGCTTCTTCT |
| COL1A1 | GCTCGTGGAAATGATGGTGC | ACCCTGGGGACCTTCAGAG |
| β-actin | GGCTGTATTCCCCTCCATCG | CCAGTTGGTAACAATGCCATG |

**Table S2. The information of primary antibodies utilized for Western blotting**

| Primary antibodies | Catalog Number | Manufacturer | Dilution Ratio |
| --- | --- | --- | --- |
| OPN | GB112328 | Servicebio | 1:500 |
| RUNX2 | GB115751 | Servicebio | 1:500 |
| BMP2 | GB15252 | Servicebio | 1:1000 |
| ACAN | 68350-1-Ig | Proteintech | 1:500 |
| PRG4 | ER1912-53 | HUABIO | 1:500 |
| SOX9 | ET1611-56 | HUABIO | 1:5000 |
| COL2A1 | HA722733 | HUABIO | 1:1000 |
| ADAMTS1 | GB111011 | Servicebio | 1:500 |
| ADAMTS5 | DF13268 | Affinity | 1:500 |
| MMP3 | GB11131 | Servicebio | 1:500 |
| MMP13 | 18165-1-AP | Proteintech | 1:1000 |
| MMP9 | GB11132 | Servicebio | 1:500 |
| ALP | DF6225 | Affinity | 1:1000 |
| OPG | R1608-4 | HUABIO | 1:1000 |
| OCN | DF12303 | Affinity | 1:500 |
| Osx | DF7731 | Affinity | 1:1000 |
| COL1A1 | HA722517 | HUABIO | 1:1000 |
| PKA | #4782 | CST | 1:1000 |
| p-PKA | #5661 | CST | 1:1000 |
| CREB | #9197 | CST | 1:1000 |
| p-CREB | #9198 | CST | 1:1000 |
| β-actin | R1207-1 | HUABIO | 1:15000 |


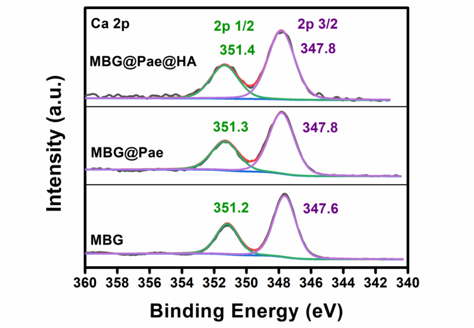


**Figure S1**. Ca2p high-resolution XPS spectrum of MBG, MBG@Pae, and MBG@Pae@HA.


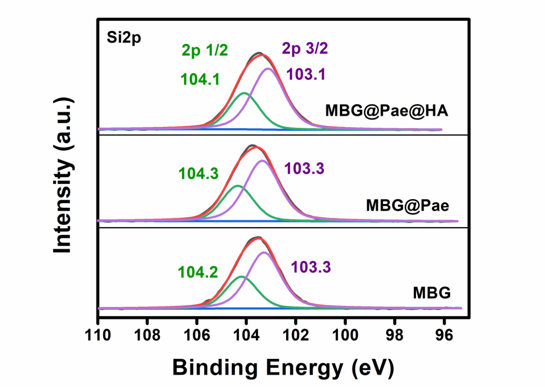


**Figure S2**. Si2p high-resolution XPS spectrum of MBG, MBG@Pae, and MBG@Pae@HA.


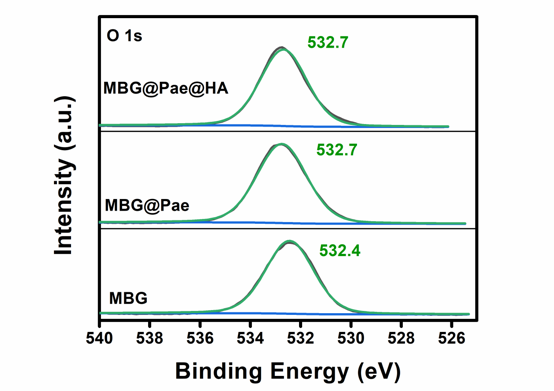


**Figure S3**. O1s high-resolution XPS spectrum of MBG, MBG@Pae, and MBG@Pae@HA.


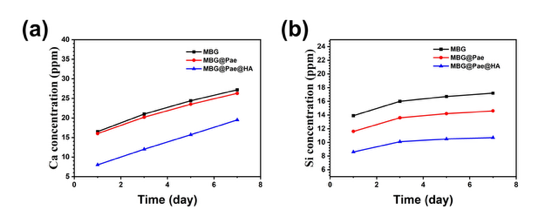


**Figure S4**. Release behaviors of (a) Ca and (b) Si ions from MBG, MBG@Pae, and MBG@Pae@HA.


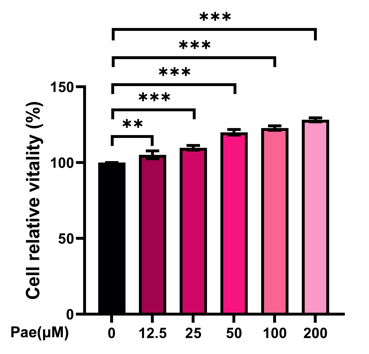


**Figure S5**. Cell viability of chondrocytes treated with free Pae.


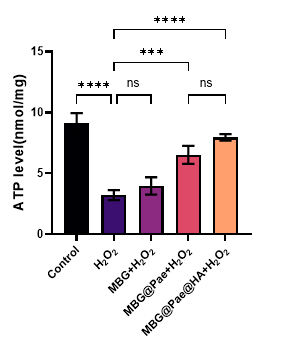


**Figure S6**. ATP levels increased by MBG@Pae@HA in H_2_O_2_-induced chondrocytes. All data are shown as the mean ± standard deviation (SD). n=5. NS: P > 0.05, *P < 0.05, **P < 0.01, and ***P < 0.001.


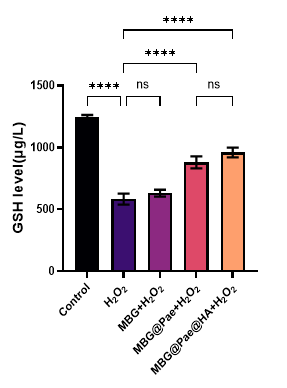


**Figure S7**. Relative expression levels of GSH increased by MBG@Pae@HA in H_2_O_2_-induced chondrocytes. All data are shown as the mean ± SD. n=5. NS: P > 0.05, *P < 0.05, **P < 0.01, and ***P < 0.001.


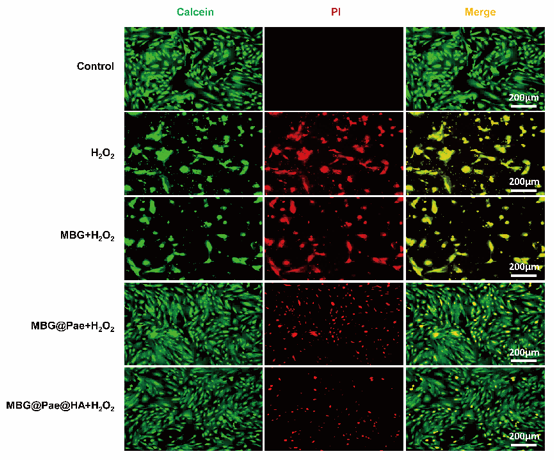


**Figure S8**. Protective effect of MBG@Pae@HA against H_2_O_2_-induced cell death in chondrocytes *via* Calcein-AM/PI double staining.


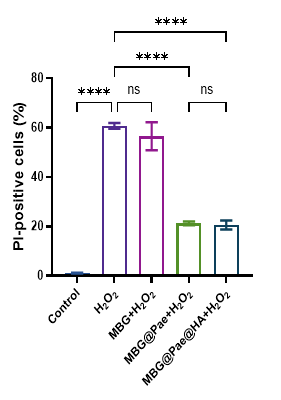


**Figure S9**. Quantification analysis of chondrocytes apoptosis after treatment with H_2_O_2_ and MBG@Pae@HA. All data are shown as the mean ± SD. n=5. NS: P > 0.05, *P < 0.05, **P < 0.01, and ***P < 0.001.


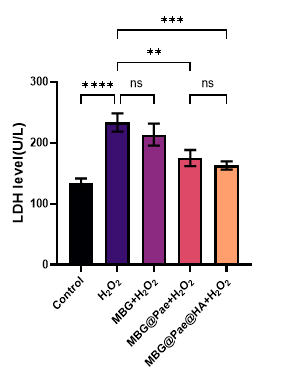


**Figure S10**. Relative expression levels of LDH reduced by MBG@Pae@HA in H_2_O_2_-induced chondrocytes. All data are shown as the mean ± SD. n=5. NS: P > 0.05, *P < 0.05, **P < 0.01, and ***P < 0.001.


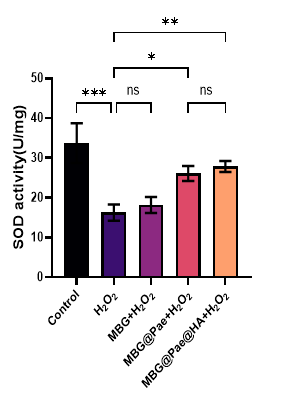


**Figure S11**. Relative SOD activity restored by MBG@Pae@HA in H_2_O_2_-induced chondrocytes. All data are shown as the mean ± SD. n=5. NS: P > 0.05, *P < 0.05, **P < 0.01, and ***P < 0.001.


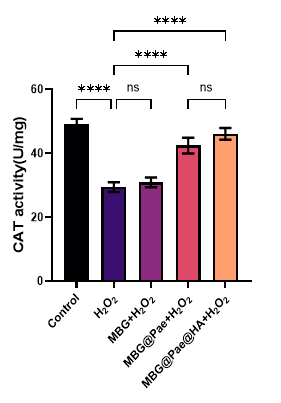


**Figure S12**. CAT activity enhanced by MBG@Pae@HA in H_2_O_2_-induced chondrocytes. All data are shown as the mean ± SD. n=5. NS: P > 0.05, *P < 0.05, **P < 0.01, and ***P < 0.001.


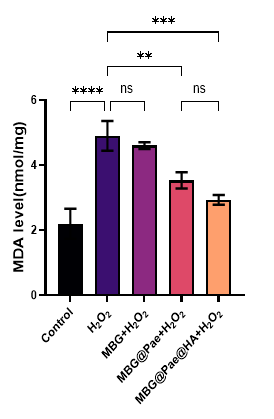


**Figure S13**. Cellular MDA levels reduced by MBG@Pae@HA in H_2_O_2_-induced chondrocytes. All data are shown as the mean ± SD. n=5. NS: P > 0.05, *P < 0.05, **P < 0.01, and ***P < 0.001.


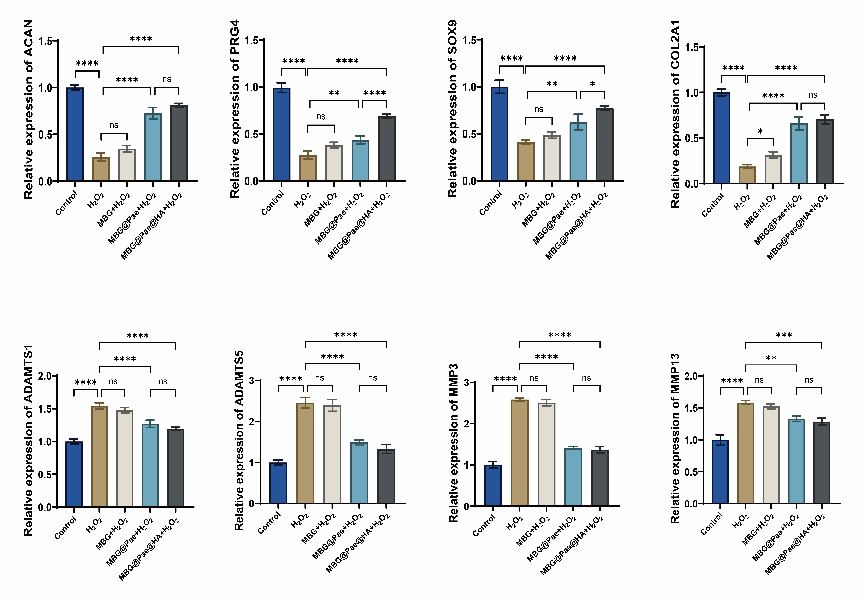


**Figure S14**. Quantitative analysis of WB results of chondrocytes


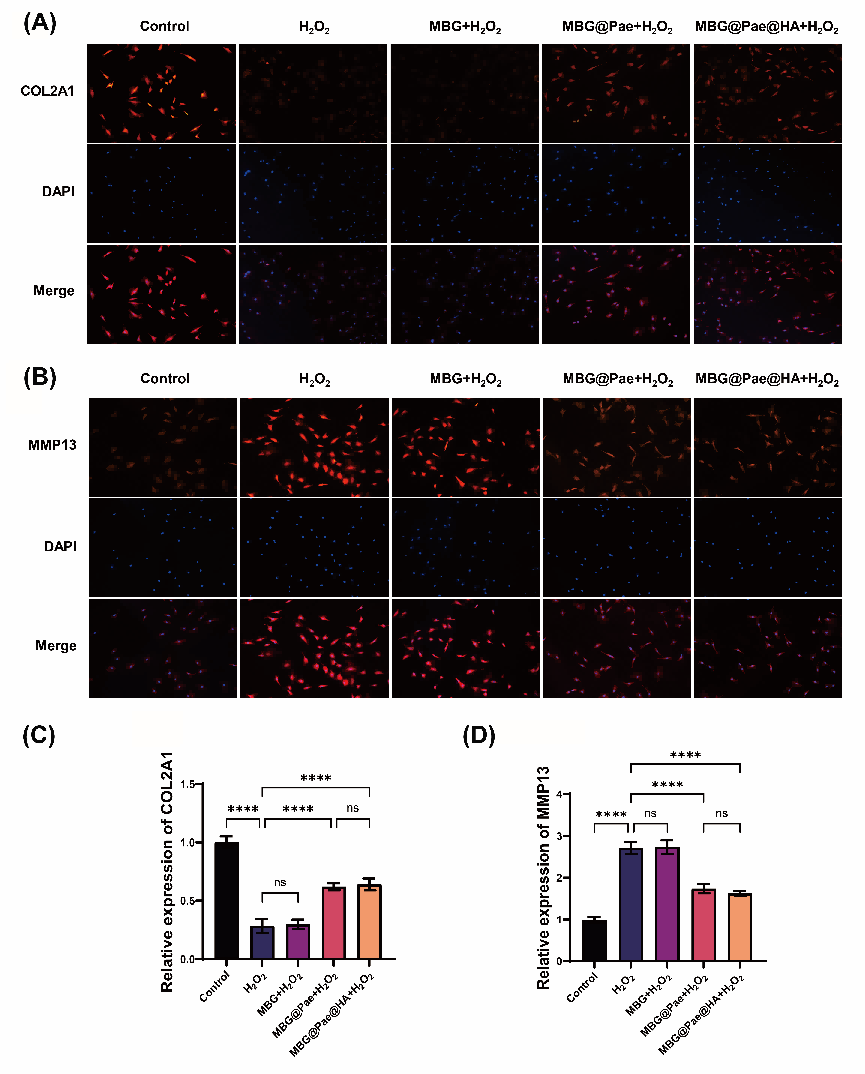


**Figure S15**. Immunofluorescence staining of COL2A1 (A, C) and MMP13 (B, D) on chondrocyte.


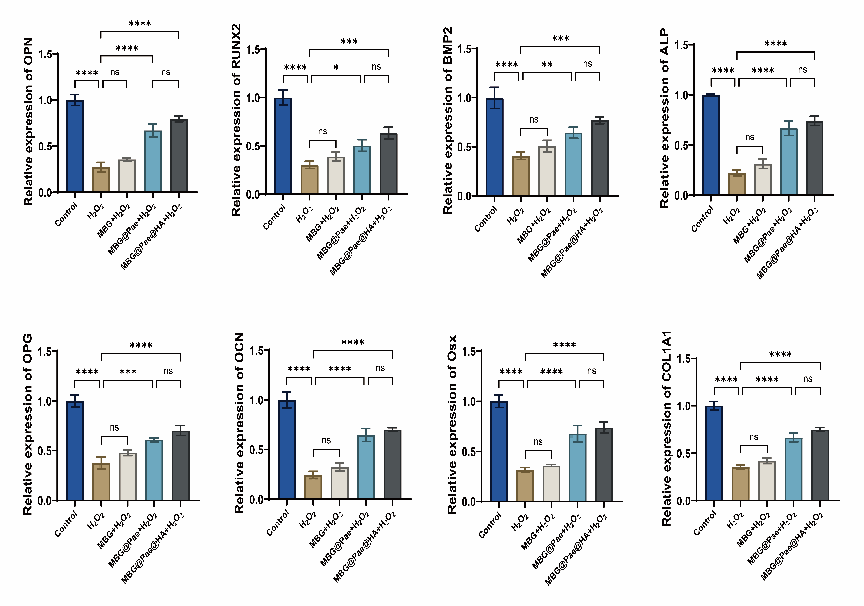


**Figure S16**. Quantitative analysis of WB results of BMSCs


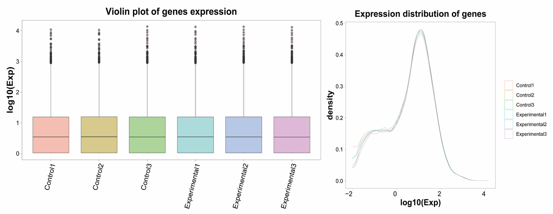


**Figure S17**. Distribution plots depicting sample distributions across groups.


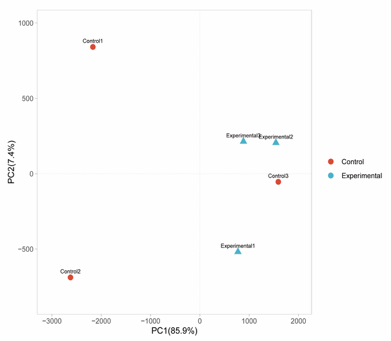


**Figure S18**. PCA plots depicting sample distributions across groups.


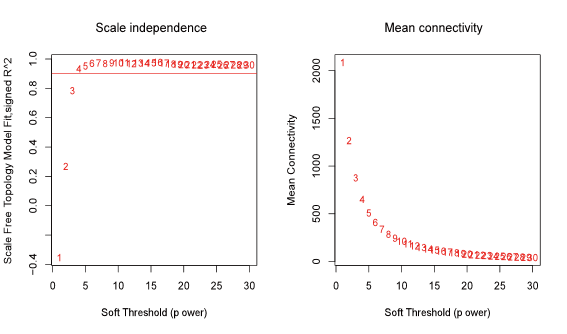


**Figure S19**. WGCNA analysis for the expression matrix of differentially expressed genes.


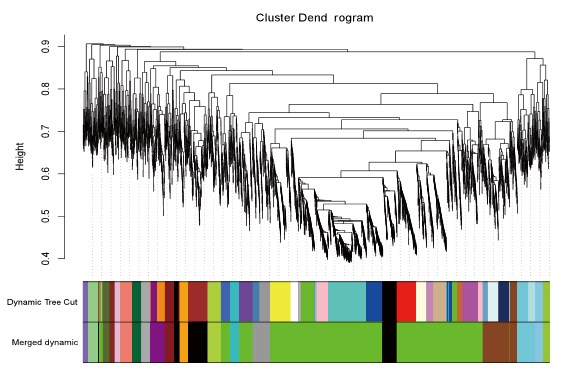


**Figure S20.** Sample clustering is conducted to detect the significant abnormal samples.


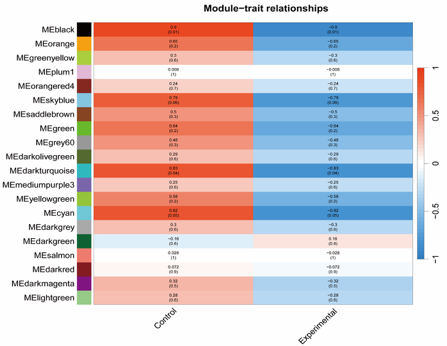


**Figure S21.** Clustering dendrogram of differentially expressed genes.


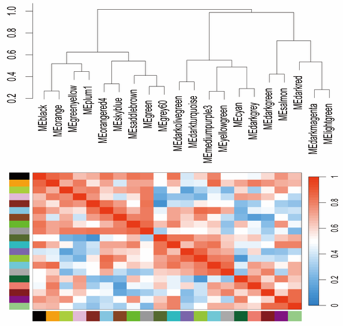


**Figure S22.** Gene expression profiles of the top 20 gene modules.

**
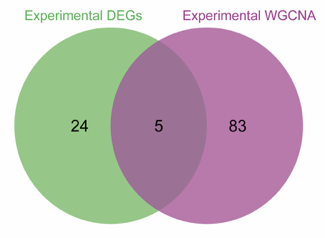
**

**Figure S23.** Venn diagram showing overlapping genes.


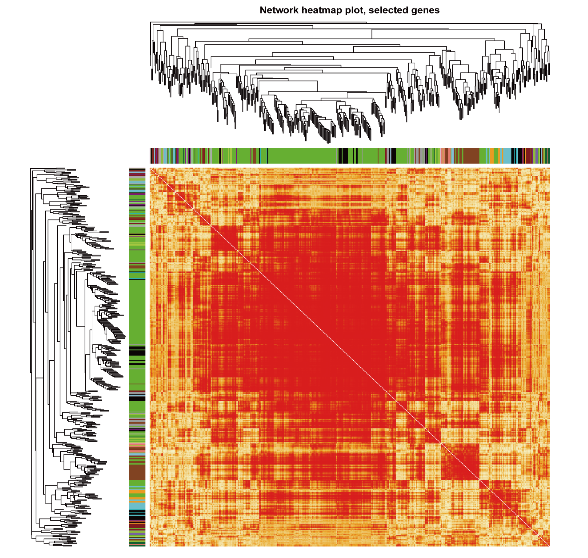


**Figure S24**. Correlation analysis for the co-expression modules of 20 genes and the expression data.


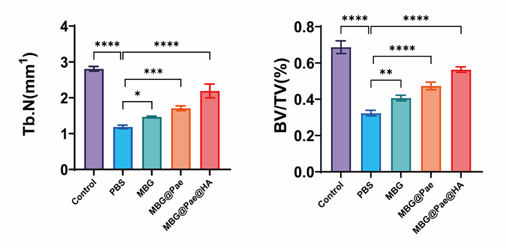


**Figure S25**. Quantitative assessment of Tb.N and BV/TV after 2 months of treatment.


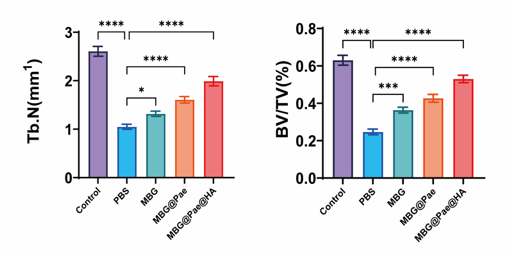


**Figure S26**. Quantitative assessment of Tb.N and BV/TV after 3 months of treatment.


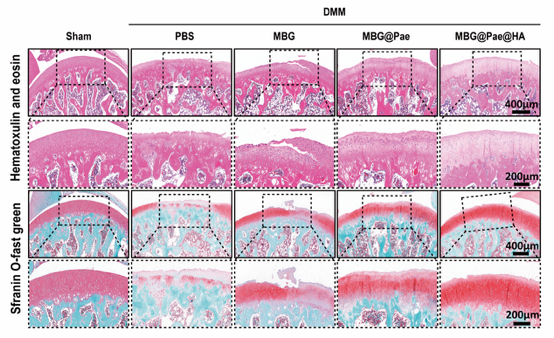


**Figure S27**. Representative images of H&E, and SO-FG staining in rat knee joints after 3 months of treatment.

**
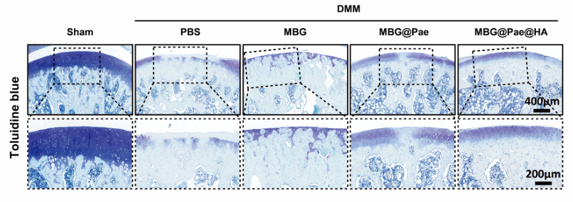
**

**Figure S28**. Representative images of TB staining in rat knee joints after 2 months of treatment.


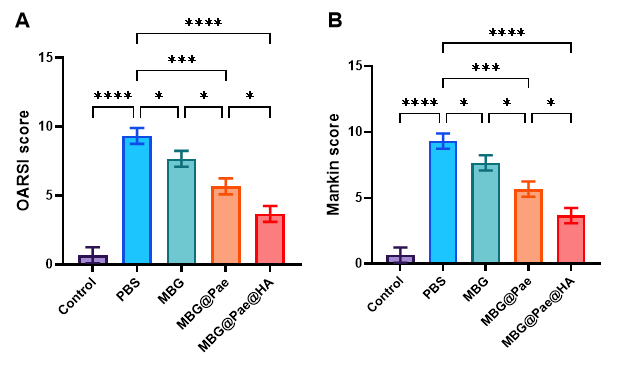


**Figure S29**. The corresponding (A) OARSI scores and (B) Mankin score after 3 months of treatment according to H&E and SO-FG staining.


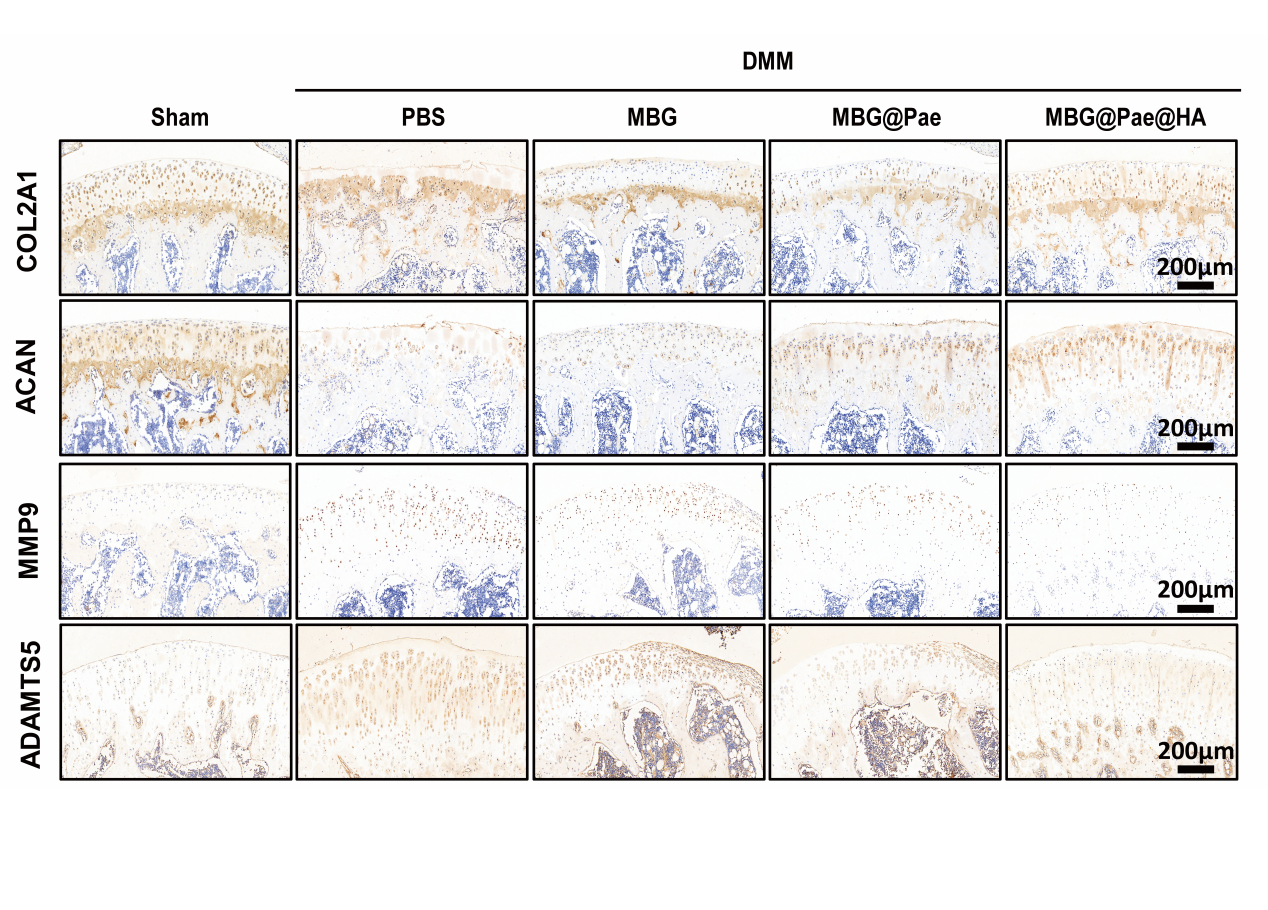


**Figure S30**. Representative images of COL2A1, ACAN, MMP9, and ADAMTS5 immunohistochemistry staining of differently treated rat knees after 3 months of treatment.


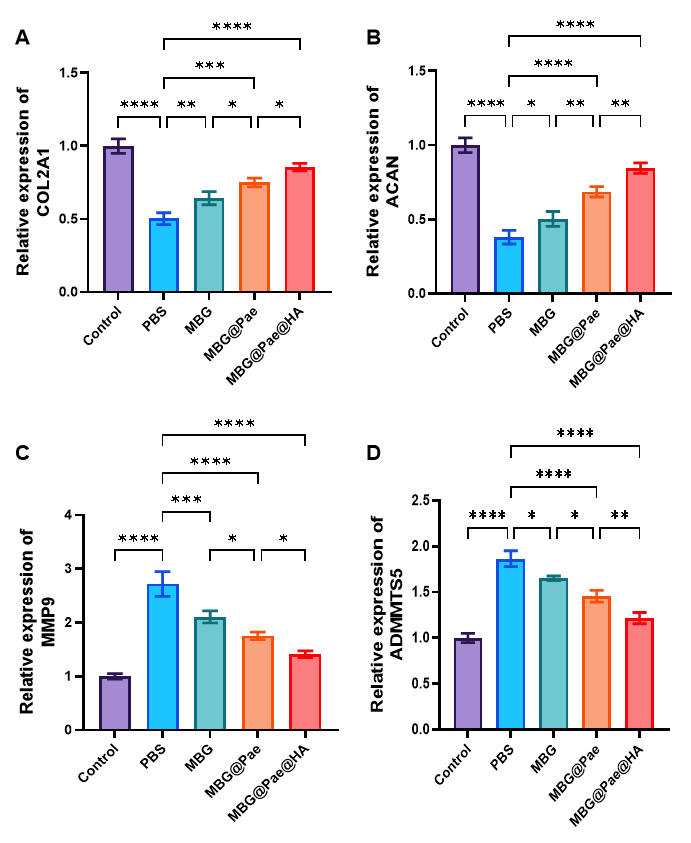


**Figure S31**. Quantitative analysis of (A) COL2A1, (B) ACAN, (C) MMP9, and (D) ADAMTS5 expression after different treatments 3 months postsurgery. All data are shown as the mean ± SD. n=3. NS: P > 0.05, *P < 0.05, **P < 0.01, and ***P < 0.001.
